# The effect of air pollution on deaths, disease burden, and life expectancy across China and its provinces, 1990–2017: an analysis for the Global Burden of Disease Study 2017

**DOI:** 10.1016/S2542-5196(20)30161-3

**Published:** 2020-08-17

**Authors:** Peng Yin, Michael Brauer, Aaron J Cohen, Haidong Wang, Jie Li, Richard T Burnett, Jeffrey D Stanaway, Kate Causey, Samantha Larson, William Godwin, Joseph Frostad, Ashley Marks, Lijun Wang, Maigeng Zhou, Christopher J L Murray

**Affiliations:** a National Center for Chronic Noncommunicable Disease Control and Prevention, Chinese Center for Disease Control and Prevention, Beijing, China; b School of Population and Public Health, The University of British Columbia, Vancouver, BC, Canada; c Institute for Health Metrics and Evaluation, University of Washington, Seattle, WA, USA; d Health Effects Institute, Boston, MA, USA; e Health Canada, Ottawa, ON, Canada

## Abstract

**Background:**

Air pollution is an important public health concern in China, with high levels of exposure to both ambient and household air pollution. To inform action at provincial levels in China, we estimated the exposure to air pollution and its effect on deaths, disease burden, and loss of life expectancy across all provinces in China from 1990 to 2017.

**Methods:**

In all 33 provinces, autonomous regions, municipalities, and special administrative regions in China, we estimated exposure to air pollution, including ambient particulate matter pollution (defined as the annual gridded concentration of PM_2·5_), household air pollution (defined as the percentage of households using solid cooking fuels and the corresponding exposure to PM_2·5_), and ozone pollution (defined as average gridded ozone concentrations). We used the methods of the Global Burden of Diseases, Injuries, and Risk Factors Study 2017 to estimate deaths and disability-adjusted life-years (DALYs) attributable to air pollution, and what the life expectancy would have been if air pollution levels had been less than the minimum level causing health loss.

**Findings:**

The average annual population-weighted PM_2·5_ exposure in China was 52·7 μg/m^3^ (95% uncertainty interval [UI] 41·0–62·8) in 2017, which is 9% lower than in 1990 (57·8 μg/m^3^, 45·0–67·0). We estimated that 1·24 million (95% UI 1·08–1·40) deaths in China were attributable to air pollution in 2017, including 851 660 (712 002–990 271) from ambient PM_2·5_ pollution, 271 089 (209 882–346 561) from household air pollution from solid fuels, and 178 187 (67 650–286 229) from ambient ozone pollution. The age-standardised DALY rate attributable to air pollution was 1513·1 per 100 000 in China in 2017, and was higher in males (1839·8 per 100 000) than in females (1198·3 per 100 000). The age-standardised death rate attributable to air pollution decreased by 60·6% (55·7–63·7) for China overall between 1990 and 2017, driven by an 85·4% (83·2–87·3) decline in household air pollution and a 12·0% (1·4–22·1) decline in ambient PM_2·5_ pollution. 40·0% of DALYs for COPD were attributable to air pollution, as were 35·6% of DALYs for lower respiratory infections, 26·1% for diabetes, 25·8% for lung cancer, 19·5% for ischaemic heart disease, and 12·8% for stroke. We estimated that if the air pollution level in China was below the minimum causing health loss, the average life expectancy would have been 1·25 years greater. The DALY rate per 100 000 attributable to air pollution varied across provinces, ranging from 482·3 (371·1–604·1) in Hong Kong to 1725·6 (720·4–2653·1) in Xinjiang for ambient pollution, and from 18·7 (9·1–34·0) in Shanghai to 1804·5 (1339·5–2270·1) in Tibet for household pollution. Although the overall mortality attributable to air pollution decreased in China between 1990 and 2017, 12 provinces showed an increasing trend during the past 27 years.

**Interpretation:**

Pollution from ambient PM_2·5_ and household burning of solid fuels decreased markedly in recent years in China, after extensive efforts to control emissions. However, PM_2·5_ concentrations still exceed the WHO Air Quality Guideline for the entire population of China, with 81% living in regions exceeding the WHO Interim Target 1, and air pollution remains an important risk factor. Sustainable development policies should be implemented and enforced to reduce the impact of air pollution on long-term economic development and population health.

**Funding:**

Bill & Melinda Gates Foundation; and China National Key Research and Development Program.

## Introduction

The subnational analyses for China from the Global Burden of Diseases, Injuries, and Risk Factors Study (GBD) 2017^1^ identified particulate matter (PM) air pollution as the fourth leading risk factor for deaths and disability-adjusted life-years (DALYs) in China in 2017. As China develops, it faces the dual challenge of exposures from both ambient PM pollution and household air pollution from solid fuels. Ambient ozone pollution is also a growing concern in China.^2^, ^3^ Although the exposure to household air pollution from solid fuels shows an overall declining trend, 32% of the Chinese population still uses solid fuels for cooking or heating.^4^ Tackling ambient PM_2·5_ pollution has become a priority for the Chinese Government,^5^ and a series of air pollution control actions have been implemented in the past few years. Reductions in PM_2·5_ concentrations have been observed in heavily polluted areas, such as Beijing.^6^ Although PM_2·5_ concentrations have started to decline in the past 5 years, absolute levels of long-term average air pollution remain high across China.^4^ Such long-term exposure is understood to have deleterious effects on public health.^7^, ^8^

**Research in context**

**Evidence before this study**

We searched PubMed for articles published up to Sept 29, 2019, using the search terms “air pollutants”, “air pollution”, “ambient particulate matter pollution”, “ozone concentration”, “PM_2·5_ exposure”, “household air pollution”, “indoor pollution”, “death”, “mortality”, “morbidity”, “DALY”, “life expectancy”, “burden”, and “China”, with the language restricted to English and Chinese. Previous evidence suggests that air pollution is a major risk factor for disease burden in China. Several previous studies have estimated the burden of ambient particulate matter and household air pollution exposure in some cities or provinces in China. However, no studies have systematically measured the variations between provinces of China in deaths and disability-adjusted life-years (DALYs) that are attributable to air pollution, and the effect of air pollution on life expectancy.

**Added value of this study**

This study measures the exposure to air pollution and its effect on deaths, DALYs, and life expectancy in every province of China in 2017 using the unified Global Burden of Diseases, Injuries, and Risk Factors Study framework. We report the effect of ambient particulate matter pollution, household air pollution from solid fuels for cooking, and ozone pollution for China and its provinces. The findings of this study show that concentrations of PM_2·5_ still exceeded the WHO Air Quality Guideline for the entire population of China in 2017, with 81% living in regions exceeding the WHO Interim Target 1. We estimated that the average life expectancy in China would have been higher by 1·25 years if the pollution levels had been lower than the minimum levels associated with health loss in 2017, including 0·84 years for ambient PM_2·5_ reduction and 0·26 years for household air pollution reduction. The effect of ambient PM_2·5_ and household air pollution on life expectancy varied considerably across provinces in China.

**Implications of all the available evidence**

Although pollution from ambient PM_2·5_ and household burning of solid fuels has dropped in recent years in China, the majority (81·1%) of the population still lives in areas exceeding the least stringent WHO Air Quality Interim Target of 35 μg/m^3^, indicating that ambient particulate matter pollution remains a challenge in China. Our study identifies areas where larger gains in life expectancy due to ambient PM_2·5_ and household air pollution reduction would be achieved, which can help to guide tailored interventions. With further economic development in China, more sustainable development policies should be instituted and enforced, to reduce the effect of air pollution on long-term economic development and population health.

Recent studies on the short-term health risks associated with ambient PM pollution and ambient ozone exposure indicate associations with deaths from cardiovascular and respiratory causes in Chinese cities.^9^, ^10^ Evidence from cohort studies in China also indicates that long-term exposure to ambient PM pollution and household air pollution from solid fuels is associated with various chronic diseases, including stroke, ischaemic heart disease, chronic obstructive pulmonary disease (COPD), and lung cancer.^11^, ^12^ The levels of air pollution exposure, background rates of cardiovascular and respiratory diseases, and population density vary widely across China. These factors are all relevant in estimating impact of air pollution in different parts of China.

In this report, we use GBD 2017 data to estimate the impact of air pollution on deaths, DALYs, and reductions in life expectancy in China and its provinces from 1990 to 2017. By including these subnational data, we present the most detailed analysis conducted to date of the health impacts of air pollution in China. This information can be used to prioritise action to address both ambient and household air pollution in China and its provinces.

## Methods

### Overview

The analysis and findings on air pollution presented in this report were part of GBD 2017. A comprehensive description of the metrics, data sources, and statistical modelling in GBD 2017 has been reported elsewhere.^1^, ^13^ The GBD 2017 methods relevant to this paper are summarised here. A detailed description of the methods is shown in appendix 2 (pp 3–14).

### Estimation of exposure to air pollution

We used the improved methods developed for GBD 2017 to estimate exposure to ambient PM_2.5_, household air pollution due to use of solid cooking fuels, and ozone. The measure of exposure to ambient PM was the average annual PM_2·5_ concentration in the air at a spatial resolution of a 0·1° × 0·1° grid cell over the globe, which is 11 × 11 km at the equator. We obtained estimates of PM_2·5_ in China from chemical transport model simulations combined with satellite retrievals of aerosol optical depth, calibrated to available ground monitoring data in a Bayesian hierarchical model.^14^ In total, 3714 measurements from 1735 unique locations in China spanning 2010–16 were used in the calibration model. Based on 20% out-of-sample evaluation, the population-weighted root mean squared error (RMSE) of the exposure estimates in China was 6 μg/m^3^. Spatial patterns estimated for 1990 reflect those from roughly the year 2000, when satellite-based estimates became available. The spatially varying calibration with ground monitoring data was applied to all years with satellite estimates, but it was based on the 2010–16 period when ground monitoring data were available. Estimates for 1990 were based on a scaling of the (calibrated) year 2000 estimates with chemical transport model simulations.

The measure of household air pollution was exposure to PM_2·5_ due to use of solid cooking fuels. GBD 2017 methods for estimating household air pollution are described elsewhere.^13^ In brief, estimates of the proportion of the population exposed were modelled using linear, spatiotemporal, and Gaussian process regression on population-based data on households using solid fuels for cooking. For each location and year, exposure to PM_2·5_ from household solid fuel use for men, women, and children was then estimated using a model mapping measurements of PM_2·5_ from studies of household solid fuel use.^15^ The concentration of ambient PM_2·5_ for each location-year was then subtracted from these exposure estimates to provide an estimate of the incremental exposure due to household solid fuel use for cooking. This approach resulted in independent estimates for PM_2·5_ exposure due to ambient PM and household solid fuel use. The major data sources to measure this use in China included the China census, China Chronic Disease and Risk Factor Surveillance, China Energy Statistical Yearbook, China Health and Nutrition Survey, China National Health Services Survey, and scientific literature (appendix 2 pp 10–11).

To match epidemiological effect estimates,^16^ ozone exposure was defined as the highest seasonal (6-month) mean daily maximum 8-h average concentration of ozone in air as parts per billion (ppb) for each 0·1° × 0·1° grid cell over the globe. The exposure to ambient ozone pollution was estimated using a continent-specific weighted blend of six chemical transport models, which was then bias-corrected at grid-cell resolution with available ground measurements, as described in detail elsewhere.^17^ China was mainly part of the east Asia domain, with small portions in the south Asia, central Asia, and Russia domains. Within the east Asia domain were a total of 1617 monitoring stations, although only 27 from China during the period covering 2008–15. The RMSE of the model blend compared with available ground measurements was 4·9 ppb within the east Asia, south Asia, and central Asia domains, and 2·0 ppb in the Russia domain. After applying bias correction, in which final estimates were set to be equal to the spatially interpolated ozone field within 2° of a ground measurement, the overall RMSE for the east Asia region reduced slightly to 4·3 ppb. Estimates of ozone exposure for 1990 were based on a similar process as that described for PM_2·5_. Chemical transport model simulations included 1990, but bias correction with ground measurements was based on the more recent periods when ground measurements were available.

### Estimation of deaths and DALYs attributable to air pollution

The GBD comparative risk assessment framework was used to estimate disease burden attributable to risk factors, as described elsewhere.^13^ In brief, the diseases linked to the different exposures followed those included in the GBD and are based on available evidence from multiple cohort studies that indicate robust associations, as described in appendix 2 (pp 6–7). We estimated the burden attributable to ambient and household air pollution for ischaemic heart disease, stroke, lung cancer, COPD, type 2 diabetes, and acute lower respiratory infections, and the burden attributable to ozone for COPD. The relative risks for mortality from these outcomes due to ambient and household air pollution were estimated using non-linear integrated exposure–response functions, which included all cohort studies published until July, 2018, including a study of mortality in a large cohort of Chinese men.^9^ Details of these GBD methods, including the input data, have been published elsewhere.^13^ The relative risk of COPD attributable to ozone was obtained from previous literature.^13^, ^18^ In addition, the burden of cataracts attributable to household air pollution was estimated for females on the basis of a standard meta-analysis approach and binary exposure to household solid fuels for cooking.^19^

For each risk factor, the theoretical minimum risk exposure level was established as the lowest level of exposure below which its relationship with a disease outcome was not supported by the available evidence. The theoretical minimum risk exposure level for ambient PM and household air pollution was defined as a uniform distribution of population-weighted mean PM_2·5_ between 2·4 and 5·9 μg/m^3^. For ozone pollution, the theoretical minimum risk exposure level was defined as a population-weighted concentration between 29·1 and 35·7 ppb.

To differentiate the disease burden from PM_2·5_ exposure due to household solid fuel use and ambient PM pollution, an updated integrated exposure–response function was used to estimate the relative risks.^13^ For populations exposed to both ambient and household air pollution, a joint relative risk was calculated from the integrated exposure–response function according to the combined level of the two exposures, and the risk attributable to each exposure was based on the proportion of the combined exposure to PM_2·5_ from each source. The purpose of this approach is to avoid potential overestimation of disease burden for individuals exposed to both forms of pollution.

Cause of death registration has rapidly expanded in China since the 1990s. Zhou and colleagues^1^ used three sources of data available at the county level to develop estimates of the age-sex-cause-specific mortality at the province level for 1990–2017. These estimates were corrected for variations in medical certification practice over time in China and for completeness of the registration. In the absence of cause of death data at a more fine-grained level within each province, we assumed that the death rates from each cause do not vary within the province. Population data for each pixel were based on World Population Prospects population estimates.^20^

### Analyses presented in this paper

We computed the attributable number of deaths and age-standardised death rate for each of the air pollution-associated causes in 2017, and we reported the percentage changes of age-standardised DALY rate from 1990 to 2017. Because the Chinese Government issued a national action plan for air pollution control in 2013,^5^ the age-standardised death rate attributable to air pollution in 2013 was also reported as a reference. We report the numbers of deaths and the death rate attributable to air pollution for five health areas in China, defined on the basis of life expectancy and mortality levels for four major cancers (stomach, lung, liver, and oesophageal), COPD, and the ratio of cerebrovascular disease to ischaemic heart disease as described in GBD 2015.^21^ Health area 1 includes six provinces with low mortality similar to high-income countries (Beijing, Hong Kong, Macau, Shanghai, Tianjin, and Shejiang); health area 2 includes six provinces with relatively high life expectancy and the lowest mortality rates of ischaemic heart disease and stroke (Fujian, Guangdong, Hainan, Hubei, Hunan, and Jiangsu); health area 3 includes 11 provinces characterised by higher than average levels of mortality due to ischaemic heart disease, stroke, and the four major cancers (Anhui, Hebei, Heilongjiang, Henan, Inner Mongolia, Jilin, Liaoning, Ningxia, Shaanxi, Shandong, and Shanxi); health area 4 includes five provinces that are mostly in the southwest of China, with lower than average life expectancy but low mortality from ischaemic heart disease and stroke and high mortality from COPD (Chongqing, Gansu, Jiangxi, Sichuan, and Yunnan); and health area 5 consists of five provinces that are mostly in the west of China (Guangxi, Guizhou, Qinghai, Tibet, and Xinjiang) with lower than average life expectancy, low mortality from ischaemic heart disease, and high mortality from COPD. We calculated the ratio of the provincial-level DALY rates to the median DALY rate among all provinces. We also analysed the decomposition of changes in mortality attributable to air pollution at the provincial level during 1990–2017. We used methods developed by Das Gupta^22^ to conduct a decomposition of changes in mortality attributable to air pollution at the provincial level in China for 1990–2017 due to population growth, population ageing, risk-deleted mortality rate (the expected mortality that would be observed if air pollution was removed), and changes in exposure to air pollution. We estimated the change in life expectancy in each province if air pollution, ambient PM_2·5_, and household air pollution concentrations had been less than the theoretical minimum risk exposure level causing health loss during 2017. We calculated estimates and 95% uncertainty intervals (UIs) for the level of exposure and cause-specific attributable deaths and DALYs based on 1000 runs of the models for each quantity of interest.

### Role of the funding source

The funder of the study had no role in study design, data collection, data analysis, data interpretation, or writing of the report. The corresponding author had full access to all the data in the study and had final responsibility for the decision to submit for publication.

## Results

**Figure 1:**
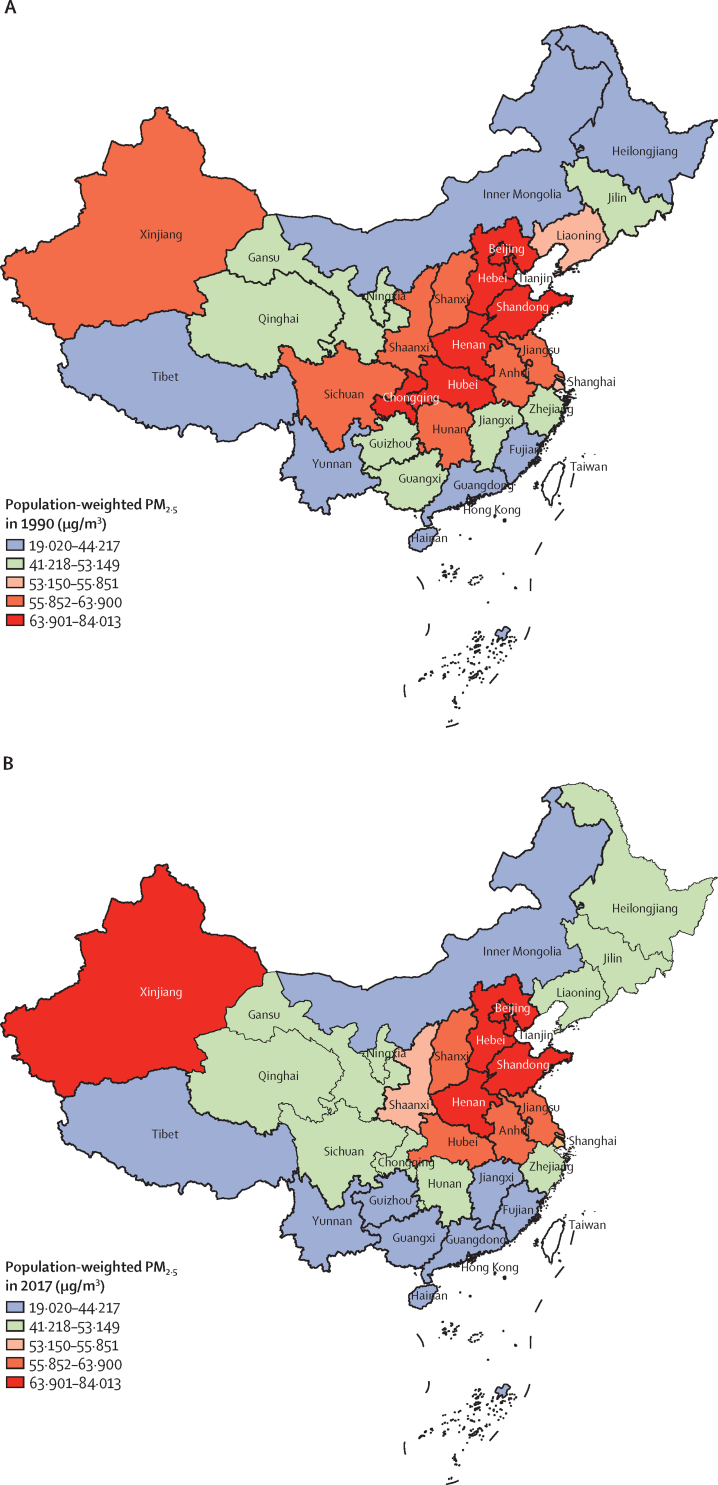
Population-weighted mean ambient PM_2·5_ concentration in provinces of China in 1990 (A) and 2017 (B)

The average annual population-weighted PM_2·5_ exposure in China was 52·7 μg/m^3^ (95% UI 41·0–62·8) in 2017, which is 9% lower than in 1990 (57·8 μg/m^3^, 45·0–67·0). The population-weighted mean annual PM_2·5_ levels in 1990 and 2017 were highest in the more populated areas in Beijing, Tianjin, Hebei and Henan, and in the more sparsely populated Xinjiang (figure 1). PM_2·5_ level increased from 1990, peaked during 2011–13, and started to decrease afterwards (appendix 2 p 16). In 2017, the entire population in China lived in areas with concentrations exceeding the 10 μg/m^3^ (annual average) WHO Air Quality Guideline, and 81·1% lived in areas with concentrations above the 35 μg/m^3^ Chinese Ambient Air Quality Standard (which is also the WHO Interim Target 1).^23^, ^24^ The proportion of the population using solid fuels for cooking in China in 2017 was 32·2%, which is significantly lower than that in 1990 (84·4%). Although a decreasing trend was seen in all provinces, the proportion of the population using solid fuels in 2017 was ten times greater in the province with the highest use (54·1% in Gansu) than in the province with the lowest use (4·9% in Shanghai; appendix 2 p 3). The annual exposure to population-weighted ozone concentration in China in 2017 was 68·2 ppb (95% UI 68·2–68·3), with the highest in Beijing (91·2 ppb, 91·0–91·5) and lowest in Macao (43·2 ppb, 42·4–44·1). The level of population-weighted annual ozone exposure remained stable at 66–68 ppb in China across 1990–2017, and the same pattern of stability was seen in all the provinces during 1990–2017 (appendix 2 p 3).

In 2017, 6·9% (95% UI 5·9–7·8) of all DALYs in China were attributable to air pollution. For specific causes, 40·0% (95% UI 29·4–49·3) of DALYs for COPD were attributable to air pollution, as were 35·6% (28·4–42·5) of DALYs for lower respiratory infections, 26·1% (17·2–30·2) for diabetes, 25·8% (19·1–32·5) for lung cancer, 19·5% (17·4–21·8) for ischaemic heart disease, and 12·8% (10·3–15·3) for stroke (appendix 2 p 18). Of the total PM_2·5_ in China, the highest proportion was in Tianjin (7·3%, 95% UI 6·3–8·5) and the lowest in Tibet (2·0%, 1·0—3·8). From the total household air pollution, the highest proportion was in Tibet (5·3%, 4·0–6·5) and the lowest in Shanghai (0·1%, 0·1–0·2; appendix 2 p 5). We estimate that 1·24 million (1·08–1·40) deaths were attributable to air pollution in China in 2017, including 851 660 (712 002–990 271) from ambient PM pollution, 271 089 (209 882–346 561) from household air pollution from solid fuels, and 178 187 (67 650–286 229) from ambient ozone pollution (table 1). The age-standardised death rate attributable to air pollution was higher in males (90·4 per 100 000, 78·4–101·9) than in females (57·1 per 100 000, 49·3–65·5).

**Table 1:** Deaths and age-standardised death rates attributable to air pollution in China in 2017

	**Air pollution**		**Ambient particulate matter pollution**		**Household air pollution from solid fuels**		**Ambient ozone pollution**	
	Deaths (thousands)	Age-standardised death rate per 100 000	Deaths (thousands)	Age-standardised death rate per 100 000	Deaths (thousands)	Age-standardised death rate per 100 000	Deaths (thousands)	Age-standardised death rate per 100 000
**All causes**	**1243·0 (1081·8–1395·4)**	**72·7 (63·2–81·5)**	**851·7 (712·0–990·3)**	**49·4 (41·2–57·5)**	**271·1 (209·9–346·6)**	**15·8 (12·2–20·2)**	**178·2 (67·7–286·2)**	**11·1 (4·2–17·8)**
Chronic obstructive pulmonary disease	429·5 (311·0–541·8)	26·8 (19·4–33·8)	227·8 (149·4–299·5)	14·2 (9·3–18·7)	81·4 (51·4–114·7)	5·0 (3·2–7·1)	178·2 (67·7–286·2)	11·1 (4·2–17·8)
Diabetes mellitus	32·6 (21·5–37·8)	1·8 (1·2–2·1)	25·0 (16·6–29·7)	1·4 (0·9–1·6)	7·6 (4·9–9·8)	0·4 (0·3–0·5)	..	..
Ischaemic heart disease	292·2 (258·0–329·9)	16·6 (14·7–18·8)	223·7 (189·2–257·7)	12·7 (10·7–14·7)	68·5 (53·3–88·6)	3·9 (3·0–5·0)	..	..
Lower respiratory infections	63·2 (49·1–82·2)	4·6 (3·6–5·9)	46·7 (35·2–63·3)	3·4 (2·5–4·5)	16·6 (12·4–22·3)	1·2 (0·9–1·6)	..	..
Stroke	246·2 (196·6–295·9)	13·5 (10·7–16·2)	186·9 (142·9–229·5)	10·2 (7·8–12·5)	59·2 (43·1–79·5)	3·2 (2·4–4·3)	..	..
Lung cancer	179·3 (131·6–224·6)	9·4 (6·9–11·8)	141·5 (102·4–181·3)	7·4 (5·4–9·5)	37·8 (25·5–51·6)	2·0 (1·3–2·7)	..	..
**Sex**								
Male	727·2 (633·7–820·0)	90·4 (78·4–101·9)	524·8 (440·8–609·8)	64·6 (53·9–75·2)	133·0 (95·7–177·9)	16·2 (11·8–21·6)	101·9 (38·5–163·5)	14·1 (5·3–22·6)
Female	515·8 (447·9–590·8)	57·1 (49·3–65·5)	326·8 (268·5–385·5)	36·1 (29·6–42·5)	138·1 (107·9–174·8)	15·2 (11·8–19·2)	76·3 (28·5–127·1)	8·7 (3·3–14·5)
**Age**								
Children (aged <5 years)	7·6 (5·9–9·4)	9·5 (7·3–11·7)	5·2 (3·8–6·7)	6·4 (4·7–8·3)	2·5 (1·8–3·2)	3·1 (2·2–4·0)	0·0 (0·0–0·0)	0·0 (0·0–0·0)
Older people (aged >70 years)	805·0 (686·1–918·8)	817·3 (696·6–932·9)	533·6 (433·8–628·8)	541·8 (440·4–638·4)	172·6 (132·1–222·1)	175·3 (134·2–225·5)	146·3 (55·9–234·7)	148·6 (56·7–238·3)

Data in parentheses are 95% uncertainty intervals.

The numbers of DALYs attributable to air pollution in 2017 in China are shown in the appendix 2 (p 19). The age-standardised DALY rate attributable to air pollution was 1513·1 per 100 000 in China in 2017, and was higher in males (1839·8 per 100 000) than in females (1198·3 per 100 000). The age-standardised death rate attributable to air pollution decreased by 60·6% (95% UI 55·7–63·7) for China overall between 1990 and 2017, with a 12·0% (1·4–22·1) decline for ambient PM pollution and an 85·4% (83·2–87·3) decline for household air pollution (appendix 2 p 20). A substantial reduction in age-standardised death rate attributable to ambient PM (8·9%, 8·6–9·0) was seen during 2013–17 in China; appendix 2 pp 16, 22).

The provinces in China showed marked variations of death rates that were attributable to air pollution. The age-standardised death rate per 100 000 that was attributable to ambient PM pollution was highest in Xinjiang (73·7, 95% UI 30·8–115·9) and Hebei (70·1, 44·0–90·6), and lowest in Hong Kong (21·4, 16·2–27·3) and Hainan (21·7, 13·9–30·7; table 2). The age-standardised death rate per 100 000 attributable to household air pollution from solid fuels was highest in Tibet (75·8, 95% UI 55·6–97·7) and lowest in Shanghai (0·8, 0·4–1·5) and Beijing (0·9, 0·4–1·6; table 2). The age-standardised death rate per 100 000 attributable to ozone pollution was highest in Tibet (23·4, 95% UI 8·6–37·7) and lowest in Hong Kong (1·3, 0·5–2·9).

**Table 2:** Deaths and age-standardised death rates attributable to air pollution, ambient particulate matter pollution, household air pollution, and ambient ozone pollution in five health areas of China in 2017

	**Air pollution**		**Ambient particulate matter pollution**		**Household air pollution from solid fuels**		**Ambient ozone pollution**	
	Deaths (thousands)	Age-standardised death rate per 100 000	Deaths (thousands)	Age-standardised death rate per 100 000	Deaths (thousands)	Age-standardised death rate per 100 000	Deaths (thousands)	Age-standardised death rate per 100 000
**China**	**1243·0 (1081·8–1395·4)**	**72·7 (63·2–81·5)**	**851·7 (712·0–990·3)**	**49·4 (41·2–57·5)**	**271·1 (209·9–346·6)**	**15·8 (12·2–20·2)**	**178·2 (67·7–286·2)**	**11·1 (4·2–17·8)**
**Health area 1**								
Shanghai	14·1 (11·2–17·5)	33·6 (26·8–41·8)	12·1 (9·5–15·1)	28·9 (22·8–36·4)	0·3 (0·1–0·6)	0·8 (0·4–1·5)	2·4 (0·9–4·0)	5·7 (2·2–9·3)
Tianjin	13·0 (10·7–15·6)	71·9 (59·7–86·3)	11·8 (9·7–14·4)	65·1 (53·4–79·0)	0·5 (0·2–0·8)	2·6 (1·3–4·5)	1·1 (0·4–1·9)	6·6 (2·5–12·0)
Zhejiang	35·3 (27·5–44·7)	45·7 (35·6–57·7)	27·4 (20·2–35·8)	35·3 (25·9–45·7)	2·9 (1·5–5·2)	3·8 (1·9–6·7)	7·0 (2·6–11·4)	9·4 (3·5–15·3)
Beijing	12·4 (10·0–15·1)	42·1 (34·0–51·1)	11·1 (8·9–13·6)	37·6 (30·1–45·6)	0·3 (0·1–0·5)	0·9 (0·4–1·6)	1·6 (0·6–2·6)	5·6 (2·3–9·4)
Hong Kong	4·2 (3·3–5·1)	27·4 (21·5–33·8)	3·3 (2·5–4·1)	21·4 (16·2–27·3)	0·8 (0·4–1·3)	5·0 (2·7–8·4)	0·2 (0·1–0·5)	1·3 (0·5–2·9)
Macau	0·2 (0·2–0·3)	30·8 (25·0–37·4)	0·2 (0·1–0·2)	23·3 (18·3–29·4)	0·0 (0·0–0·1)	5·7 (3·0–9·4)	0·0 (0·0–0·0)	2·5 (0·9–4·6)
**Health area 2**								
Jiangsu	73·8 (59·8–89·2)	63·4 (51·3–76·3)	56·3 (43·5–69·8)	47·9 (37·0–59·1)	7·4 (4·1–12·6)	6·3 (3·5–10·9)	15·0 (5·8–24·2)	13·7 (5·3–21·9)
Hainan	4·5 (3·4–5·8)	42·5 (32·4–54·6)	2·3 (1·5–3·3)	21·7 (13·9–30·7)	1·8 (1·2–2·6)	16·9 (10·8–24·2)	0·5 (0·2–0·9)	5·2 (1·8–9·0)
Guangdong	53·1 (42·2–64·9)	49·2 (39·2–60·1)	43·1 (32·6–53·8)	39·6 (30·0–49·4)	6·1 (3·5–9·9)	5·7 (3·3–9·2)	5·3 (1·9–9·1)	5·3 (1·9–9·0)
Fujian	18·5 (14·6–23·2)	42·1 (33·3–52·7)	13·5 (10·3–17·3)	30·4 (23·4–39·0)	3·1 (1·8–4·8)	7·0 (4·1–11·1)	2·6 (1·0–4·4)	6·3 (2·3–10·4)
Hubei	58·7 (47·3–71·7)	83·6 (67·6–102·0)	41·9 (31·1–53·0)	58·8 (43·5–74·5)	11·2 (6·7–17·5)	16·0 (9·5–25·0)	8·4 (3·1–13·4)	13·2 (4·9–21·0)
Hunan	76·5 (61·8–92·1)	92·7 (75·0–110·8)	50·1 (35·6–65·7)	59·9 (42·6–78·0)	18·7 (11·3–27·8)	22·8 (13·7–33·8)	11·3 (4·2–18·7)	14·7 (5·5–24·3)
**Health area 3**								
Shandong	104·4 (82·9–126·9)	78·1 (62·2–95·1)	78·9 (54·7–100·8)	58·5 (40·5–74·8)	15·8 (9·1–25·2)	11·8 (6·8–19·0)	14·8 (5·6–24·6)	11·8 (4·4–19·7)
Hebei	77·1 (60·8–94·9)	90·9 (71·7–112·8)	60·3 (38·2–78·2)	70·1 (44·0–90·6)	11·2 (5·8–19·5)	13·2 (7·0–23·1)	8·8 (3·3–15·2)	11·9 (4·5–20·9)
Ningxia	4·2 (3·4–5·1)	67·9 (55·3–82·0)	2·7 (2·0–3·5)	42·4 (31·2–54·9)	1·1 (0·7–1·6)	18·5 (12·1–26·2)	0·6 (0·2–0·9)	10·4 (4·0–16·9)
Jilin	22·4 (17·6–27·7)	61·1 (47·9–75·7)	16·9 (12·2–22·0)	45·6 (33·0–59·0)	4·8 (2·6–7·5)	13·1 (7·0–20·5)	1·1 (0·4–2·6)	3·5 (1·2–8·4)
Liaoning	47·0 (37·7–57·2)	69·7 (56·1–84·5)	38·6 (29·1–49·2)	56·8 (43·2–72·4)	6·4 (3·3–11·0)	9·5 (5·0–16·3)	2·9 (1·1–6·2)	4·9 (1·9–10·4)
Shanxi	27·3 (21·6–34·1)	63·6 (50·1–79·4)	18·8 (13·4–24·9)	43·2 (30·6–57·1)	6·4 (4·0–9·7)	14·8 (9·2–22·6)	3·3 (1·3–5·7)	8·4 (3·3–14·4)
Shaanxi	29·9 (23·8–37·5)	62·3 (50·0–77·9)	20·1 (13·8–27·4)	41·4 (28·4–56·2)	7·7 (4·8–11·5)	16·1 (10·1–24·0)	3·2 (1·3–5·4)	7·2 (2·8–12·0)
Henan	95·4 (78·8–115·8)	88·8 (73·6–107·1)	68·9 (51·8–87·6)	63·5 (47·8–80·8)	20·1 (12·2–31·2)	18·8 (11·6–29·1)	10·0 (3·9–17·8)	10·3 (3·9–18·6)
Anhui	56·4 (45·6–69·9)	68·3 (55·6–84·8)	36·5 (25·9–47·4)	43·7 (31·0–56·6)	14·5 (9·6–20·9)	17·5 (11·6–25·3)	8·3 (3·1–13·9)	10·6 (4·0–17·7)
Inner Mongolia	21·3 (16·8–26·1)	77·3 (61·4–94·4)	13·0 (8·0–18·1)	46·1 (28·2–64·1)	6·7 (3·8–10·1)	24·2 (13·9–36·5)	2·4 (0·9–4·0)	10·2 (3·9–17·1)
Heilongjiang	39·2 (29·8–50·0)	78·1 (59·2–98·4)	28·2 (17·9–39·6)	55·4 (35·6–77·7)	9·3 (5·2–14·7)	18·6 (10·3–29·3)	2·5 (0·9–4·9)	6·0 (2·1–11·9)
**Health area 4**								
Jiangxi	36·1 (29·7–43·4)	76·6 (62·9–91·6)	20·1 (14·5–26·4)	42·0 (30·1–55·0)	12·6 (8·4–17·9)	26·8 (17·9–37·9)	5·0 (1·8–8·3)	11·4 (4·3–19·0)
Chongqing	33·6 (25·7–42·2)	73·0 (56·0–91·5)	21·2 (15·1–28·4)	46·1 (33·0–61·6)	7·3 (4·6–10·8)	16·0 (10·1–23·5)	7·5 (2·7–12·2)	16·2 (5·9–26·4)
Yunnan	43·1 (33·8–52·2)	89·8 (70·2–109·0)	19·6 (12·5–27·9)	40·2 (25·7–57·1)	18·7 (13·1–25·5)	39·0 (27·0–53·2)	6·8 (2·5–11·4)	14·9 (5·4–25·1)
Gansu	26·9 (21·8–32·6)	97·0 (78·1–117·4)	12·4 (8·2–17·2)	44·1 (28·9–61·0)	10·8 (7·6–14·6)	38·8 (27·6–52·6)	5·6 (2·1–9·1)	21·3 (7·9–34·9)
Sichuan	108·7 (83·2–133·8)	91·0 (70·0–112·0)	63·7 (35·7–86·8)	52·9 (29·2–71·3)	27·9 (17·4–42·6)	23·4 (14·8–35·5)	25·4 (9·7–41·2)	21·8 (8·4–35·0)
**Health area 5**								
Tibet	2·6 (2·0–3·1)	120·9 (95·4–149·3)	0·6 (0·3–1·2)	29·3 (14·1–55·0)	1·6 (1·2–2·1)	75·8 (55·6–97·7)	0·4 (0·2–0·7)	23·4 (8·6–37·7)
Xinjiang	21·9 (15·8–28·0)	112·1 (80·5–143·1)	14·7 (6·1–23·1)	73·7 (30·8–115·9)	5·3 (2·5–9·2)	26·7 (12·7–46·8)	3·0 (1·1–4·9)	17·9 (6·2–29·4)
Qinghai	5·2 (4·0–6·3)	101·4 (79·7–123·5)	2·8 (1·5–3·8)	54·0 (28·5–73·1)	1·7 (1·1–2·5)	32·8 (22·0–48·1)	1·0 (0·4–1·6)	21·5 (8·2–35·1)
Guangxi	41·0 (33·1–49·8)	72·6 (58·8–87·9)	24·7 (17·9–32·7)	43·4 (31·5–57·5)	13·2 (9·0–18·7)	23·5 (16·0–33·1)	4·5 (1·6–7·5)	8·3 (3·0–13·8)
Guizhou	35·0 (28·3–42·2)	88·4 (71·9–106·3)	16·1 (11·3–22·3)	40·2 (28·3–55·0)	15·0 (10·6–20·3)	38·1 (26·7–51·0)	5·6 (2·1–9·5)	14·8 (5·5–24·7)

Data in parentheses are 95% uncertainty intervals. Health areas are defined in the Methods.^21^

**Figure 2:**
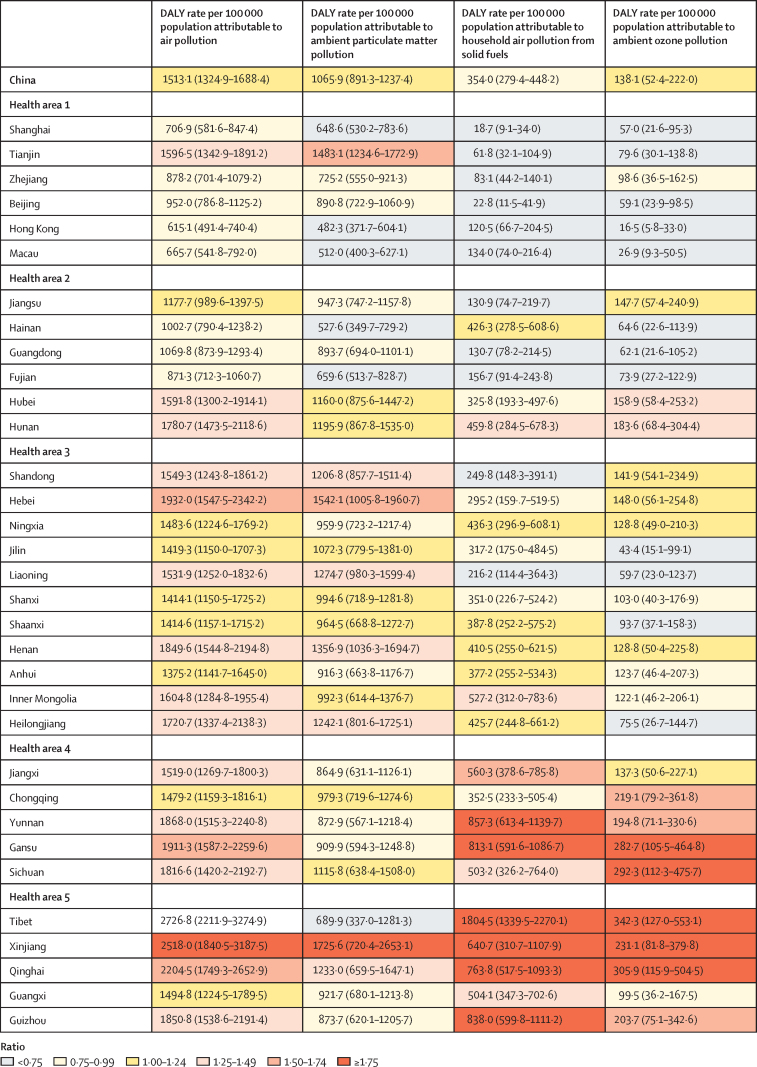
DALY rates attributable to air pollution, ambient particulate matter pollution, household air pollution, and ambient ozone pollution in the provinces of China, 2017 Data are n (95% uncertainty interval). Coloured key depicts the ratio of the provincial age-standardised DALY rates to the median age-standardised DALY rate for all of the provinces. Health areas are defined in the Methods.^21^ DALY=disability-adjusted life-year.

The DALY rate attributable to household air pollution from solid fuels was highest in health areas 4 and 5, mostly in western and under-developed provinces, compared with the other health areas in China (figure 2). In health area 1, Tianjin had a substantially higher DALY rate attributable to ambient PM pollution than the national average (figure 2). The DALY rate per 100 000 population attributable to overall air pollution was highest in Tibet (2726·8, 95% UI 2211·9–3274·9), Xinjiang (2518·0, 1840·5–3187·5), and Qinghai (2204·5, 1749·3–2652·9), and lowest in Hong Kong (615·1, 491·4–740·4), Macau (665·7, 541·8–792·0), and Shanghai (706·9, 581·6–847·4).

**Figure 3:**
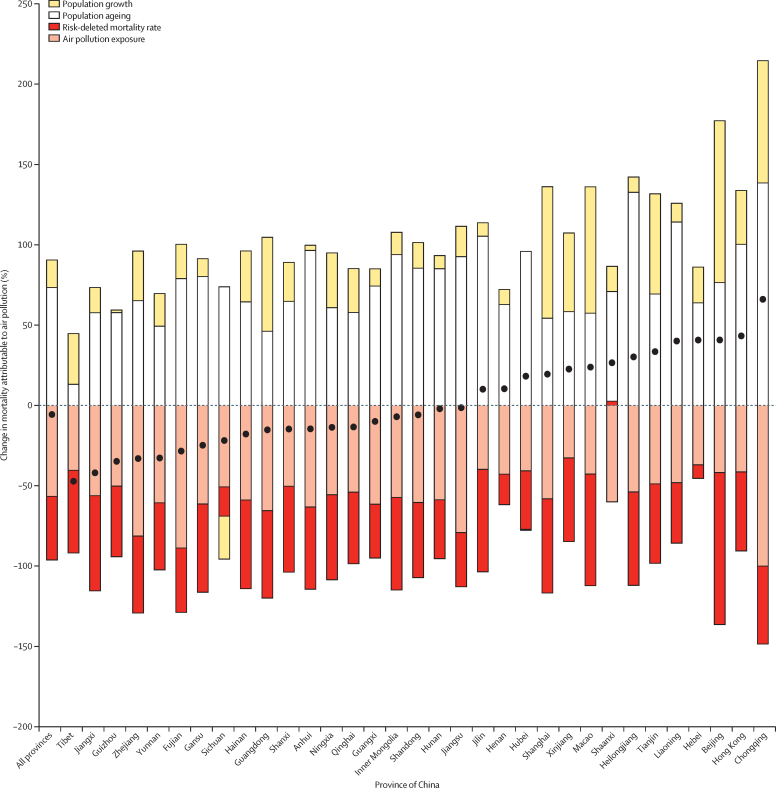
Changes in mortality attributable to air pollution from 1990 to 2017 by province in China, according to the contributions of population growth, population ageing, risk-deleted mortality rate, and air pollution exposure Black dots indicate overall change in mortality.

Concerning the changes in mortality attributable to air pollution from 1990 to 2017, the overall age-standardised death rate attributable to air pollution decreased with declining air pollution exposure and air pollution-deleted mortality, which offset increasing population growth and population ageing in China from 1990 to 2017 (figure 3). In Chongqing, Liaoning, Heilongjiang, and Hong Kong, there are higher amounts of population ageing compared with other provinces and population growth offset reductions in exposure and mortality rate, leading to a net increase in attributable burden.

**Figure 4:**
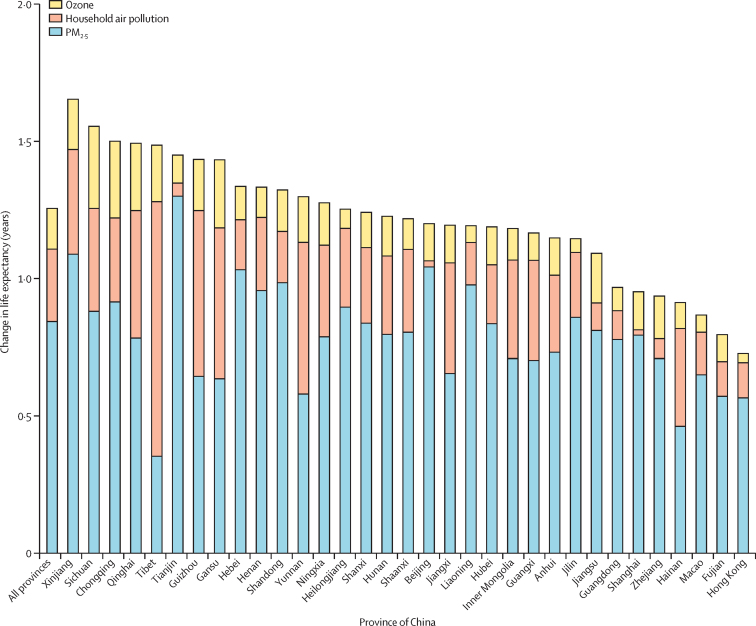
Changes in life expectancy by province in China in 2017 if air pollution had been lowered to the theoretical minimum risk exposure levels

If air pollution had been lowered to the theoretical minimum risk exposure levels for the year 2017, then the average life expectancy change in China in 2017 would have been 1·25 years, with the largest effect in Xinjiang (1·65 years) and the smallest in Hong Kong (0·73 years; figure 4). If the exposure to ambient PM_2·5_ had been lower than the minimum risk exposure levels, life expectancy would have increased in China by 0·84 years, with the largest change in Tianjin (1·30 years) and the lowest in Tibet (0·35 years; figure 4). For household air pollution from solid fuels, the life expectancy change in China was 0·26 years, with the largest gain in Tibet (0·93 years) and lowest in Shanghai (0·02 years; figure 4). For ambient ozone pollution, the life expectancy change was 0·15 years, with the largest gain in Sichuan (0·30 years) and lowest in Hong Kong (0·03 years).

## Discussion

China has faced the challenge of household air pollution from use of solid fuels for a long time, particularly in its western provinces. With rapid economic development and urbanisation during the past two decades, household air pollution has decreased while ambient air pollution has become a leading risk factor in China, affecting in particular the large population living in urban areas. It is noteworthy that the Chinese Government recognised this serious problem and took comprehensive actions to tackle air pollution.^5^ The reduction of air pollution exposure levels and decreased mortality attributable to air pollution during the past 28 years observed in our analysis might reflect these efforts: for ambient PM pollution, the age-standardised death rate fell between 1990 and 2017, with the greatest reductions seen for household air pollution from solid fuels. More pronounced decreases were seen from 2013 to 2017. A recent study^25^ also reported the same declining trend of age-standardised deaths attributable to PM_2·5_ pollution.

In China, PM_2·5_ pollution has dropped in recent years with the implementation of a series of national and regional control measures. The State Council issued a National Air Pollution Prevention and Control Action Plan^5^ in 2013, with ten detailed strategies and measures for implementation, including all related government agencies. Targets were set to reduce PM_2·5_ concentrations in highly polluted areas, such as a 25% reduction in Beijing, Tianjin, and Hebei provinces, 20% reduction in the Yangtze River Delta, which includes Shanghai, Jiangsu, Zhejiang, and Anhui provinces, and 15% reduction in the Pearl River Delta in Guangdong province. Although a systematic evaluation of these measures is not yet available, a 2018 analysis^6^ of air quality and related health effects in 74 key Chinese cities found that annual average PM_2·5_ concentrations decreased by 33·3% between 2013 and 2017. However, the majority (81·1%) of the population still lives in areas exceeding the least stringent WHO air quality interim target of 35 μg/m^3^, indicating that ambient PM pollution remains a challenge in China.

Various studies have explored the main contributors to PM_2·5_ in China.^26^, ^27^ The most recent Global Burden of Disease from Major Air Pollution Sources study^28^ reported that coal combustion from industry, electricity, and domestic sources was the most important contributor to ambient PM_2·5_, causing 40% of population-weighted PM_2·5_ in China in 2013. Other important sources of PM_2·5_ emission in China include transportation, domestic biomass, and non-coal industrial and open burning. *World Urbanization Prospects*,^29^ published by the UN, reported that between 2018 and 2050 China is projected to add another 25 million urban dwellers, with over 80% of the population living in urban areas by 2050. The rapid pace of urbanisation in China presents a considerable environmental challenge. The adaptation of an integrated and sustainable urban planning strategy is a paramount priority for central and local government officials.

Our study found a substantial reduction of the DALY rate attributable to household air pollution in China and all provinces. The proportion of households cooking with solid fuels in China has decreased from 61% in 2005 to 32% in 2017, largely due to the aggressive efforts to reduce household burning of coal for cooking and heating. The government banned the use of coal for household cooking and heating in regions around Beijing, in favour of a switch to natural gas, and has promoted clean energy to replace solid fuels across the country.

The DALYs attributable to household air pollution varied by a factor of 55 across the provinces in China, suggesting serious disparities in terms of socioeconomic development, disease burden, and risk factor control between Chinese provinces. Tibet had the highest burden due to household air pollution, but its burden from ambient PM was the lowest in China. The classification of five health areas in China, as discussed in GBD 2013, was based on life expectancy, mortality of cancers, cardiovascular diseases, and respiratory diseases.^21^ The pattern observed for these factors was similar for DALYs attributable to household air pollution and ozone, with the higher burden in health areas 4 and 5. The burden caused by ambient PM, however, did not follow the same pattern. Tianjin, in health area 1, had higher DALYs attributable to PM_2·5_, and provinces from each health area showed mixed results due to substantial variations of both exposure levels and mortality rates.

Although ambient PM and household air pollution have been widely recognised as important contributors to disease burden in China, ozone has received less attention. We report that ozone pollution is also an important risk factor for deaths and DALYs from chronic respiratory diseases. Associations between ambient ozone pollution and increased mortality from cardiovascular diseases have also been reported in the Chinese population, but a recent systematic review of current evidence^30^ does not support a causal relationship between long-term exposure to ozone and mortality from cardiovascular diseases or lung cancer.^30^ A 2018 study^6^ found that although PM_2·5_, PM_10_, sulphur dioxide, and carbon monoxide all decreased between 2013 and 2017, the annual average concentration of ozone remained unchanged in the same period.^6^ In addition to the current efforts on PM control, reducing the emission of nitrogen oxides and volatile organic compounds, and increasing public awareness of the harmful effects caused by ozone should be strengthened in China.

We estimated that the average life expectancy in China would have been higher by 1·25 years if the pollution levels had been lower than the minimum levels associated with health loss in 2017, including 0·84 years for ambient PM_2·5_ reduction and 0·26 years for household air pollution reduction. The effect of PM_2·5_ on life expectancy varied by a factor of four across the provinces in China. The potential gains in life expectancy due to household air pollution reduction were even more variable, with considerably larger potential benefits in western provinces than in eastern provinces—eg, Tibet had a potential increase that was 46 times higher than that in Shanghai. The estimated effect of air pollution on loss of life expectancy at birth from our study is considerably different from those provided in the studies by Chen and colleagues^31^ and Ebenstein and colleagues.^32^ This difference comes from both the sources of data and the methodology used to derive the effect of air pollution. In their 2013 study, Chen and colleagues^31^ took advantage of a unique national policy in China to assess the effect of the total suspended particles on population health, as represented by life expectancy at birth. This study was updated with an assessment of the effect of PM_10_ in 2017.^32^ These studies used mortality data from the Chinese Disease Surveillance Points for 1991–2000 and 2004–12, whereas our study used the most recent and complete mortality dataset in China. Methodologically, the age and cause pattern of mortality should also be considered in estimates of the effect of air pollution on life expectancy, given that child mortality went through a rapid decline in China in the 1990s and that mortality levels for those younger than 5 years are less affected by air pollution while having a huge effect on life expectancy at birth. In addition, these studies assume a linear relationship between exposure and life expectancy,^31^, ^32^ whereas our estimates use the non-linear integrated exposure–response function, which indicates reduced marginal benefits at higher exposure levels.^33^, ^34^

This analysis has some important limitations. First, although the integrated exposure–response risk function was updated to include one large-scale cohort study from China that assessed the effects of long-term exposure to PM_2·5_, the integrated exposure–response still mainly relies on studies of other combustion sources to estimate PM_2·5_ relative risks at high levels of exposure, which is a source of uncertainty in the overall estimates.^11^, ^35^ Second, ground monitoring of PM_2·5_ was not widely available in China until 2013, and satellite-based estimates only began in 1998. Estimates of PM_2·5_ for previous years are therefore more uncertain. A similar limitation applies to ozone estimates because the bias correction with ground measurements only reflects more recent years. Third, disease burden was estimated at the province level, but considerable variation probably exists across counties within a province. Fourth, when evaluating the disease burden attributable to household air pollution, we only included household solid fuel use for cooking but not for heating. Previous studies have reported that both heating and cooking could contribute to premature deaths in China,^36^ and the absence of estimates on household solid fuel use for heating might bias our results. Fifth, the diseases linked to the different exposures might under-represent the overall effect of these exposures because emerging research might indicate that additional diseases are associated with these risk factors. Sixth, we assumed independent effects of exposure to ambient PM_2·5_ and household air pollution. We estimated the burden attributable to household air pollution by evaluating the incremental increase in exposure above that from the time-specific and location-specific ambient PM_2·5_ levels. However, given that epidemiological studies have not evaluated joint effects, risk additivity might not apply and multiplicative or more complex interactions might occur.^37^ Furthermore, other possible pollutants such as nitrogen dioxide were not included, and exposure to PM_2·5_ and ozone might not be sufficient to fully characterise the toxicity of the atmospheric mix or to fully account for the risk of mortality associated with exposure to ambient pollution.^38^

To conclude, pollution from ambient PM_2·5_ and the household burning of solid fuels has dropped substantially in recent years in China, after extensive efforts to control emissions. However, PM_2·5_ concentrations still exceed the WHO Air Quality Guideline for the entire population of China, with 81% of people living in regions exceeding the WHO Interim Target 1. The Chinese Government issued the Healthy China Action 2019–2030^39^ in July 2019, and healthy environment promotion is one of the 15 major areas for action, but recent increases in Chinese coal burning capacity will pose challenges to continuing progress in reducing air pollution levels and disease burden, as well as meeting climate change goals.^40^ The environmental Kuznets curve^41^ predicts that environmental pollution will follow an inverse U-shaped curve with respect to economic growth; economic development entails rising levels of environmental pollution, which improves after some level of income growth has occurred.^42^ China's experience has, unfortunately, corroborated the first stage of this model. It is in everyone's interest that, with further economic development in China, more sustainable development polices will be instituted and enforced to reduce the effect of air pollution on long-term economic development and population health.

## Data sharing

To download GBD data used in the analyses in this Article please visit the Global Health Data Exchange GBD 2017 website.

## Supplementary Materials

Chinese translation of the abstract
