# Supplementary material for: The effect of air pollution on deaths, disease burden, and life expectancy across China and its provinces, 1990–2017: an analysis for the Global Burden of Disease Study 2017
Source: Lancet Planet Health. 2020 Aug 17;4(9):e386–98. doi: 10.1016/S2542-5196(20)30161-3 (PMC7487771; doi:10.1016/S2542-5196(20)30161-3)
Supplement: Chinese translation of the abstract [file mmc1.pdf]

THE LANCET
Planetary Health

Supplementary appendix

This translation in Chinese was submitted by the authors and we reproduce it as supplied. It has not been peer reviewed. *The Lancet*'s editorial processes have only been applied to the original in English, which should serve as reference for this manuscript.

此简体中文译文由作者提交，我方按照提供的版本刊登。此译文并未经过同行审阅。医学期刊《柳叶刀》的编辑流程仅适用于英文原稿，英文原稿应作为此手稿的参考。

Supplement to: Yin P, Brauer M, Cohen AJ, et al. The effect of air pollution on deaths, disease burden, and life expectancy across China and its provinces, 1990–2017: an analysis for the Global Burden of Disease Study 2017. *Lancet Planet Health* 2020; published online Aug 17. http://dx.doi.org/10.1016/S2542-5196(20)30161-3.

摘要

背景：大气污染在中国是一个重要的公共卫生问题。为了对中国及各省的大气污染进行政策干预，我们估算了 1990 至 2017 年中国及各省大气污染所致的死亡、疾病负担和期望寿命损失。

方法：在中国 33 个省、自治区、直辖市和特别行政区，我们估算了大气污染水平，包括细颗粒物($PM_{2.5}$年平均浓度)，室内空气污染(室内使用固体燃料做饭的比例和相应 $PM_{2.5}$的暴露水平)和臭氧(年平均臭氧浓度)。我们采用全球疾病负担 2017 的方法估计归因于大气污染的伤残调整寿命年(Disability-adjusted life-years, DALY)，以及大气污染对人群期望寿命的影响。

结果：2017 年中国人群加权的 $PM_{2.5}$暴露水平为 52.7μg/m^3(95%置信区间 41.0μg/m^3-62.8μg/m^3)，比 1990 年(57.8μg/m^3, 45.0μg/m^3-67.0μg/m^3)低 9%。2017 年，中国有 124 万人死于大气污染 (95%置信区间：108 万-140 万)，有 851,700 (712,000-990,300)人死于室外 $PM_{2.5}$暴露，271,100（209,900-346,000）人死于室内固体燃料燃烧所致空气污染，178,200 (67,700-286,000)人死于臭氧污染。2017 年，中国归因于大气污染的年龄标化 DALY 率为 1513.1/100,000，男性(1839.8/100,000)高于女性(1198.3/100,000)。从 1990 年至 2017 年，归因于大气污染的年龄别死亡率下降 60.6% (55.7%-63.7%)，归因于室内空气污染的年龄别死亡率下降 85.4%(83.2%-87.3%)，归因于室外 $PM_{2.5}$的年龄别死亡率下降 12.0%(1.4%-22.1%)。慢性阻塞性肺病的 DALY 有 40.0%归因于大气污染，缺血性心脏病和脑卒中的 DALY 分别有 19.5%和 12.8%归因于大气污染。如果中国的大气污染降低到可导致健康损失的最低水平，人群期望寿命可增加 1.25 岁。归因于大气污染的每十万人 DALY 率在不同省份之间均有差异，例如归因于室外空气污染的 DALY 率最低为香港(482.3, 371.1-604.1), 最高为新疆(1725.6, 720.4-2653.1)，归因于室内空气污染的 DALY 率最低为上海(18.7, 9.1-34.0)，最高为西藏(1804.5, 1339.5-2270.1)。尽管 1990 至 2017 年间中国大气污染所致的总死亡率下降，但其中仍有 12 个省份呈现上升趋势。

解读：中国在控制污染物排放上做出了大量的努力，因此近年来室外 $PM_{2.5}$和室内固体燃料燃烧所致的大气污染水平明显下降。然而，中国 $PM_{2.5}$浓度仍然超过 WHO 的空气质量指南中的建议浓度，仍有 81%的人口居住在 $PM_{2.5}$浓度超过 WHO 第一级目标水平的区域，因此大气污染仍然是一个重要的危险因素。应实施和加强可持续发展政策，以减少大气污染对长期经济发展和人群健康的影响。
