# Supplementary material for: The effect of air pollution on deaths, disease burden, and life expectancy across China and its provinces, 1990–2017: an analysis for the Global Burden of Disease Study 2017
Source: Lancet Planet Health. 2020 Aug 17;4(9):e386–98. doi: 10.1016/S2542-5196(20)30161-3 (PMC7487771; doi:10.1016/S2542-5196(20)30161-3)
Supplement: Supplementary appendix 2 [file mmc2.pdf]

THE LANCET
Planetary Health

Supplementary appendix

This appendix formed part of the original submission and has been peer reviewed. We post it as supplied by the authors.

Supplement to: Yin P, Brauer M, Cohen AJ, et al. The effect of air pollution on deaths, disease burden, and life expectancy across China and its provinces, 1990–2017: an analysis for the Global Burden of Disease Study 2017. *Lancet Planet Health* 2020; published online Aug 17. http://dx.doi.org/10.1016/S2542-5196(20)30161-3.

The impact of air pollution on deaths, DALYs and life expectancy in China and its provinces, 1990-2017: the Global Burden of Disease Study 2017

Peng Yin[1], Michael Brauer[2,3], Aaron J Cohen[3,4], Haidong Wang[3], Jie Li[1], Richard T. Burnett[5], Jeffrey D Stanaway[3], Kate Causey[3], Samantha Larson[3], William Godwin[3], Joseph Frostad[3], Ashley Marks[3], Lijun Wang[1], Maigeng Zhou[1*], Christopher JL Murray[3]

[1]National Center for Chronic Noncommunicable Disease Control and Prevention, Chinese Center for Disease Control and Prevention, Beijing, China
[2]School of Population and Public Health, The University of British Columbia, Vancouver, British Columbia, Canada
[3]Institute for Health Metrics and Evaluation, University of Washington, Seattle, Washington, USA
[4]Health Effects Institute, Boston, Massachusetts, USA
[5]Health Canada, Ottawa, Ontario, Canada

*Corresponding to Dr. Maigeng Zhou, Email: zhoumaigeng@ncncd.chinacdc.cn

Web Appendix

Table of contents

1. GBD 2017 air pollution estimation methods 3
2. Use of solid fuels in provinces of China, 1990 and 2017; population weighted mean ambient ozone in provinces of China, 1990 and 2017. 15
3. Population weighted mean $PM_{2.5}$ levels, age-standardized death rate and DALY rate due to ambient $PM_{2.5}$ in China from 1990 to 2017. 16
4. Population Attributable Fractions (%) of DALYs for air pollution in all provinces in China, 2017 17
5. Percentage and 95% UI of DALYs attributable to air pollution for different diseases in China, 2017 18
6. DALYs (in thousand) attributable to air pollution with 95% uncertainty interval of China in 2017 19
7. Age-standardized death rate and DALY rate attributable to air pollution of China, 1990 and 2017 with percentage changes 20
8. Comparison of age-standardized death rate and DALY rate attributable to air pollution in 2013 and 2017 in China 22

1. GBD 2017 air pollution estimation methods

The materials presented here are adapted from:
GBD 2017 Risk Factors Collaborators. Global, regional, and national comparative risk assessment of 84 behavioural, environmental and occupational, and metabolic risks or clusters of risks for 195 countries and territories, 1990–2017: a systematic analysis for the Global Burden of Disease Study 2017. *Lancet* 2018; 2018; 392: 1923–94.

A. GBD estimation process for risk factors including air pollution

The approach used in GBD 2017 for comparative risk assessment to estimate population attributable fractions for risk factors is shown in the following flowchart.

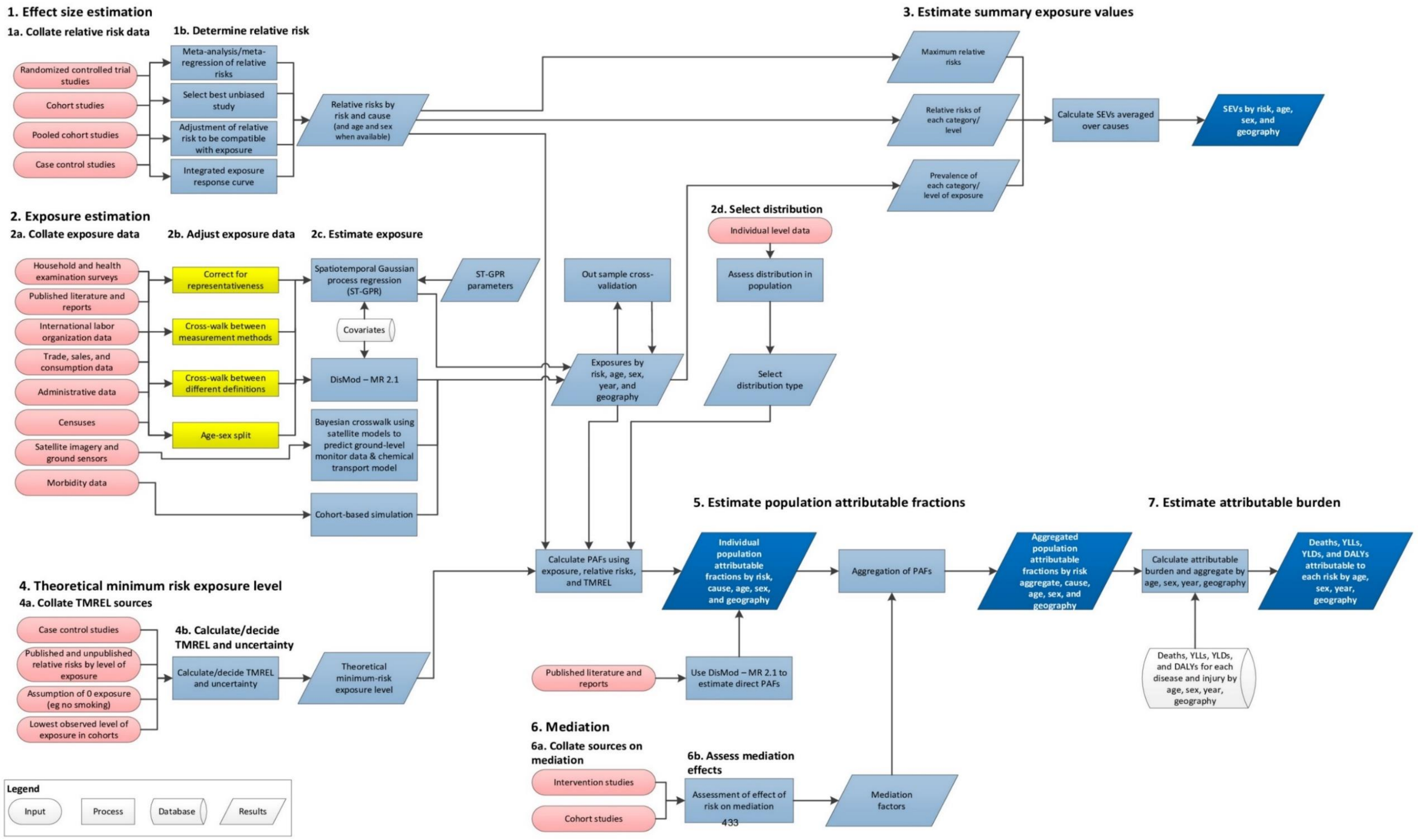

GBD is Global Burden of Disease. SEV is summary exposure value. TMREL is theoretical minimum-risk exposure level. PAF is population attributable fraction. YLL is years of life lost. YLD is years lived with disability. DALY is disability-adjusted life-year. Ovals represent data inputs, rectangular boxes represent analytical steps, cylinders represent databases, and parallelograms represent intermediate and final results.

Ambient Particulate Matter Pollution

The steps in the estimation of disease burden attributable to ambient particulate matter pollution are shown in the following flowchart:

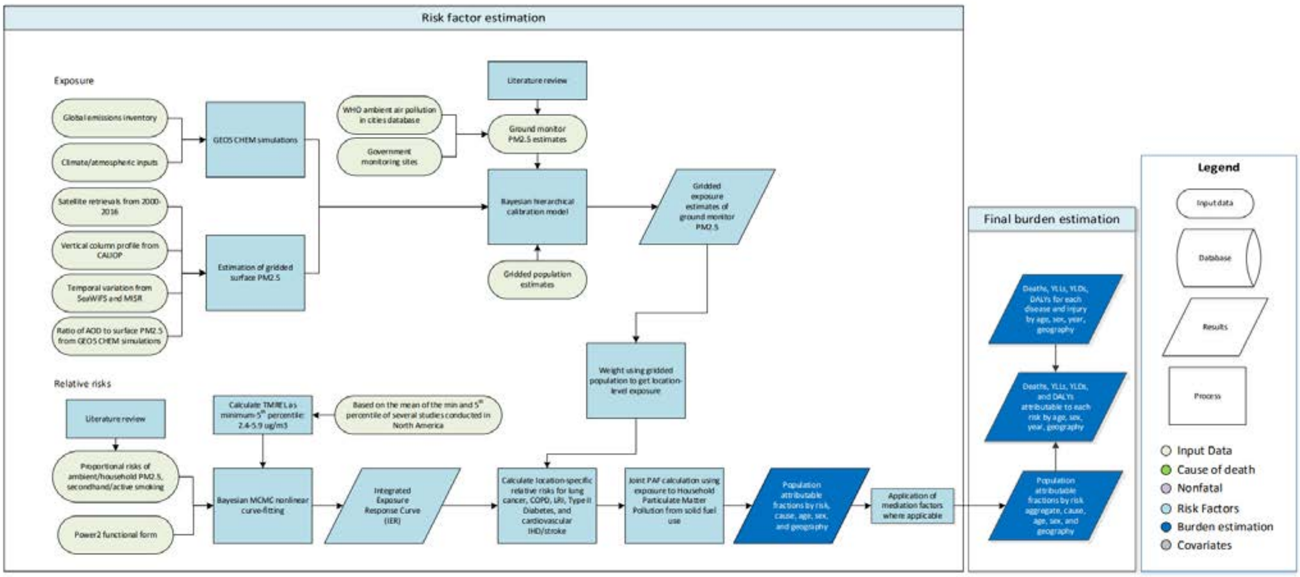

Exposure definition

Exposure to ambient air pollution is defined as the population-weighted annual average mass concentration of particles with an aerodynamic diameter less than 2.5 micrometers ($PM_{2.5}$) in a cubic meter of air. This measurement is reported in µg/m^3.

Input Data

The data used to estimate exposure to ambient air pollution is drawn from multiple sources, including satellite observations of aerosols in the atmosphere, ground measurements, chemical transport model simulations, population estimates, and land-use data.

$PM_{2.5}$ ground measurement database

Updates of ground measurements used for GBD 2017 include using more recent data than that used previously and the addition of data from new locations. The data from the 2018 update of the WHO Global Ambient Air Quality Database include monitor-specific measurements of concentrations of PM_{10} and $PM_{2.5}$ from 9,960 ground monitors (up from 6,003 in GBD 2016) from 108 countries. The majority of measurements were recorded in 2016 (as there is a lag in reporting measurements, little data from 2017 were available). Annual averages were excluded if they were based on less than 75% coverage within a year. Collection year ranged from 2008 to 2017 in data used. If information on coverage was not available then data were included unless they were already sufficient data within a country (monitor density greater than 0.1).

Satellite-based estimates

The updated satellite-based estimates for years 1998-2016 are described in detail in van Donkelaar et al. 2016.1 These estimates were available at 0.1º ×0.1º resolution (~11 x 11 km resolution at the equator) and combine aerosol optical depth retrievals from multiple satellites with the GEOS Chem chemical transport model and land use information.

Chemical transport model simulations

Estimates of the sum of particulate sulfate, nitrate, ammonium and organic carbon and the compositional concentrations of mineral dust simulated using the GEOS Chem chemical transport model, and a measure combining elevation and the distance to the nearest urban land surface (as described in van Donkelaar et al. 2016[1]) were available for 2000 to 2016 for each 0.1^o ×0.1^o grid cell. These were not included within the GBD 2013 analysis.

Modelling strategy

Significant advances have been made in the methodology used to estimate exposure to ambient particulate matter pollution since GBD 2013. The following is a summary of the modelling approach, known as the Data Integration Model for Air Quality (DIMAQ) used in GBD 2015, 2016, and 2017; further details can be found in Shaddick et al.[2] In GBD 2010 and GBD 2013 exposure estimates were obtained using a single global function to calibrate available ground measurements to a 'fused' estimate of $PM_{2.5}$; the mean of satellite-based estimates and those from the TM5 chemical transport model, calculated for each 0.1^o ×0.1^o grid cell. This was recognised to represent a trade-off between accuracy and computationally efficiency when utilising all the available data sources. In particular, the GBD 2013 exposure estimates were known to underestimate ground measurements in specific locations (see discussion in Brauer et al.[3]). This underestimation was largely due to the use of a single, global, calibration function, whereas in reality the relationship between ground measurements and other variables will vary spatially.

In GBD 2015 and GBD 2016, coefficients in the calibration model were estimated for each country. Where data were insufficient within a country, information can be `borrowed' from a higher aggregation (region) and if enough information is still not available from an even higher level (superregion). Individual country level estimates were therefore based on a combination of information from the country, its region and super-region. This was implemented within a Bayesian Hierarchical modelling (BHM) framework. BHMs provide an extremely useful and flexible framework in which to model complex relationships and dependencies in data. Uncertainty can also be propagated through the model allowing uncertainty arising from different components, both data sources and models, to be incorporated within estimates of uncertainty associated with the final estimates. The results of the modelling comprise a posterior distribution for each grid cell, rather than just a single point estimate, allowing a variety of summaries to be calculated. The primary outputs here are the median and 95% credible intervals for each grid cell. Based on the availability of ground measurement data, modelling and evaluation was focused on the year 2016.

The GBD 2017 model was updated to also include within country calibration variation.[4] The model used for GBD 2017, henceforth referred to as DIMAQ2, provides a number of substantial improvements over the initial formulation of DIMAQ. In DIMAQ, ground measurements from different years were all assumed to have been made in the primary year of interest (i.e. 2014 for GBD 2015 before extrapolation) and then regressed against values from other inputs (e.g. satellites etc.) made in that year. In the presence of changes over time therefore, and particularly in areas where no recent measurements were available, there was the possibility of mismatches between the ground measurements and other variables. In DIMAQ2, ground measurements and matched with other inputs (over time) and the possibility of the (global level) coefficients being allowed to vary over time, subject to smoothing that is induced by a second-order random walk process. In addition, the manner in which

spatial variation can be incorporated within the model has developed: where there is sufficient data, the calibration equations can now vary (smoothly) both within and between countries, achieved by allowing the coefficients to follow (smooth) Gaussian processes. Where there is insufficient data within a country, to produce accurate equations, as before information is borrowed from lower down the hierarchy and it is supplemented with information from the wider region.

Population-weighted exposure generation

To generate a distribution of the population-weighted ambient particulate matter, we took a weighted sampling strategy, taking samples from all grid cells in a given location. For example, for a country with n grid cells, we randomly sampled 1000 values from the n (grid cells) x 1000 (samples) where the probability of being sampled was proportional to the population of that grid cell.

Theoretical minimum-risk exposure level

The TMREL was assigned a uniform distribution with lower/upper bounds given by the average of the minimum and 5th percentiles of outdoor air pollution cohort studies exposure distributions conducted in North America, with the assumption that current evidence was insufficient to precisely characterise the shape of the concentration-response function below the 5th percentile of the exposure distributions. The TMREL was defined as a uniform distribution rather than a fixed value in order to represent the uncertainty regarding the level at which the scientific evidence was consistent with adverse effects of exposure. The specific outdoor air pollution cohort studies selected for this averaging were based on the criteria that their 5th percentiles were less than that of the American Cancer Society Cancer Prevention II (CPSII) cohort's 5th percentile of 8.2 based on Turner et al.[5] This criterion was selected since GBD 2010 used the minimum, 5.8, and 5th percentile solely from the CPS II cohort. The resulting lower/upper bounds of the distribution for GBD 2017 were 2.4 and 5.9. This has not changed since GBD 2015.

Relative risks and population attributable fractions

We estimated the Ambient Air Pollution-attributable burden of disease based on the relation of long-term exposure to $PM_{2.5}$ with Ischemic Heart Disease, stroke, COPD, lung cancer and acute lower respiratory infection. These were also the pollutant-outcome pairs used to estimate the Ambient Air Pollution attributable burden since GBD 2010. For GBD 2017 we also added Type II Diabetes as an outcome of ambient air pollution. We used results from all cohort studies published as of July 2018 that reported cause-specific relative risk estimates based on measured or modelled $PM_{2.5}$ and that adjusted for potential confounding due to other major risk factors such as tobacco smoking using data for each study participant.

Bowe et al. recently published work that assembled the evidence for the relationship between particulate matter and diabetes to generate IER curves and attributable burden estimates based on methodologies similar to those of the GBD.[6]

Integrated exposure response function

The Integrated Exposure Response Function (IER) was created to ascertain the shape of the dose response curve for a variety of health outcomes across a wide range of exposure to $PM_{2.5}$. The IER model is fit by integrating RR information from studies of outdoor air pollution (OAP), Second hand tobacco smoke (SHS), Household Air Pollution (HAP), and Active Smoking (AS). Because OAP

studies are often performed at the lower end of the ambient air pollution range, incorporating other exposures to particulate matter enables RR estimation across the global range of exposure. These methods have been described in detail elsewhere.[7,8]

Notable changes for GBD 2017 include added studies for OAP, SHS, and HAP, updated literature reviews for AS studies, and more informative priors to stabilize the shape of the IER curves.

-We added all newly published cohorts of long-term exposure to Ambient $PM_{2.5}$ and incidence or mortality due to IHD, stroke, COPD, lung cancer, and LRI. One notable addition was the China Male Cohort which included mortality due to IHD, Stroke, COPD, Lung Cancer, and Diabetes (unpublished analysis).[9] his study represented a higher exposure range than most of our previously incorporated studies with 5th and 95th percentile of 15.5 and 77.1 micrograms/m^3 . For Type II Diabetes, the new outcome included in GBD 2017, we included all cohorts which measured long-term $PM_{2.5}$ exposure and incident diabetes or mortality due to diabetes.

-We did not change the SHS input studies with the exception of including all studies from a recent meta-analysis examining the relationship between SHS and Type II Diabetes.[10] We also added seven studies found from a systematic review examining SHS exposure and COPD. We had previously not included SHS in the formation of this curve.

-We added four cohort studies of HAP and any of our measured outcomes. Previously we have only included which measured levels of $PM_{2.5}$ exposure. To incorporate cohort studies with binary exposure data (presence or absence of solid-fuel use for cooking) we used the $PM_{2.5}$ mapping function (see Household Air Pollution section for more details) to obtain a $PM_{2.5}$ level attributed to solid fuel use for cooking for the location-year of the study (ExpHAP). We also used the OAP exposure model to obtain an OAP $PM_{2.5}$ level for the location-year (ExpOAP). The study RR was used to inform the curve on the range of ExpOAP to (ExpOAP + ExpHAP).

-For all outcomes, we used updated systematic reviews of the literature performed by the GBD smoking team for studies examining cigarettes smoked per day and the six IER outcomes to inform the high exposure range of the curve. The smoking team found that the process of systematic review and inclusion of all acceptable studies led to lower relative risks. All citations for studies can be found using the GBD 2017 Data Input Sources Tool.

-To help obtain more reasonable curve fits, we added more informative priors to two of three IER function parameters in the MCMC Bayesian fitting process.

References

1. van Donkelaar, A.; Martin, R. V; Brauer, M.; Hsu, N. C.; Kahn, R. A.; Levy, R. C.; Lyapustin, A.; Sayer, A. M.; Winker, D. M. Global Estimates of Fine Particulate Matter using a Combined Geophysical-Statistical Method with Information from Satellites, Models, and Monitors. Environ. Sci. Technol. 2016, 50 (7), 3762–3772

2. Shaddick, G., Thomas, M.L., Jobling, A., Brauer, M., van Donkelaar, A., Burnett, R., Chang, H., Cohen, A., Van Dingenen, R., Dora, C. and Gumy, S., 2016. Data Integration Model for Air Quality: A Hierarchical Approach to the Global Estimation of Exposures to Ambient Air Pollution. Journal of Royal Statistical Society Series C (Applied Statistics). 2017. DOI: 10.1111/rssc.12227

3. Brauer, M.; Freedman, G.; Frostad, J.; van Donkelaar, A.; Martin, R. V; Dentener, F.; Van Dingenen, R.; Estep, K.; Amini, H.; Apte, J. S.; et al. Ambient Air Pollution Exposure Estimation for the Global Burden of Disease 2013. Environ. Sci. Technol. 2015, 50 (1), 79–88.

4. Shaddick G, Thomas M, Amini H, Broday DM, Cohen A, Frostad J, Green A, Gumy S, Liu Y, Martin RV, Prüss-Üstün A, Simpson D, van Donkelaar A, Brauer M. Data integration for the assessment of population exposure to ambient air pollution for global burden of disease assessment. Environ Sci Technol. 2018 Jun 29. doi: 10.1021/acs.est.8b02864
5. Turner MC, Jerrett M, Pope CA 3rd, Krewski D, Gapstur SM, Diver WR, Beckerman BS, Marshall JD, Su J, Crouse DL, Burnett RT. Long-term ozone exposure and mortality in a large prospective study . Am J Respir Crit Care Med. 2016; 193(10): 1134-42.
6. Bowe B, Xie Y, Li T, Yan Y, Xian H, Al-Aly Z. The 2016 global and national burden of diabetes mellitus attributable to PM2.5 air pollution. The Lancet Planetary Health. 2018; 2(7): e301– 12.
7. Cohen AJ, Brauer M, Burnett R, et al. Estimates and 25-year trends of the global burden of disease attributable to ambient air pollution: an analysis of data from the Global Burden of Diseases Study 2015. Lancet 2017; published online April 10. http://dx.doi.org/10.1016/S0140-6736(17)30505-6.
8 Burnett RT, Pope CA 3rd, Ezzati M, Olives C, Lim SS, Mehta S, Shin HH, Singh G, Hubbell B, Brauer M, Anderson HR, Smith KR, Balmes JR, Bruce NG, Kan H, Laden F, Prüss-Ustün A, Turner MC, Gapstur SM, Diver WR, Cohen A. An integrated risk function for estimating the global burden of disease attributable to ambient fine particulate matter exposure. Environ Health Perspect. 2014; 122(4): 397-403.
9. Yin P, Brauer M, Cohen A, Burnett RT, Liu J, Liu Y, Liang R, Wang W, Qi J, Wang L, Zhou M. Long-term Fine Particulate Matter Exposure and Nonaccidental and Cause-specific Mortality in a Large National Cohort of Chinese Men. Environ Health Perspect. 2017; 125(11): 117002.
10. Xiaomin Wei, Meng E., Sufang Yu. A meta-analysis of passive smoking and risk of developing Type 2 Diabetes Mellitus. Diabetes Research and Clinical Practice. 2015; 107(1): 9-14.

Household Air Pollution

The steps in the estimation of disease burden attributable to household air pollution are shown in the following flowchart:

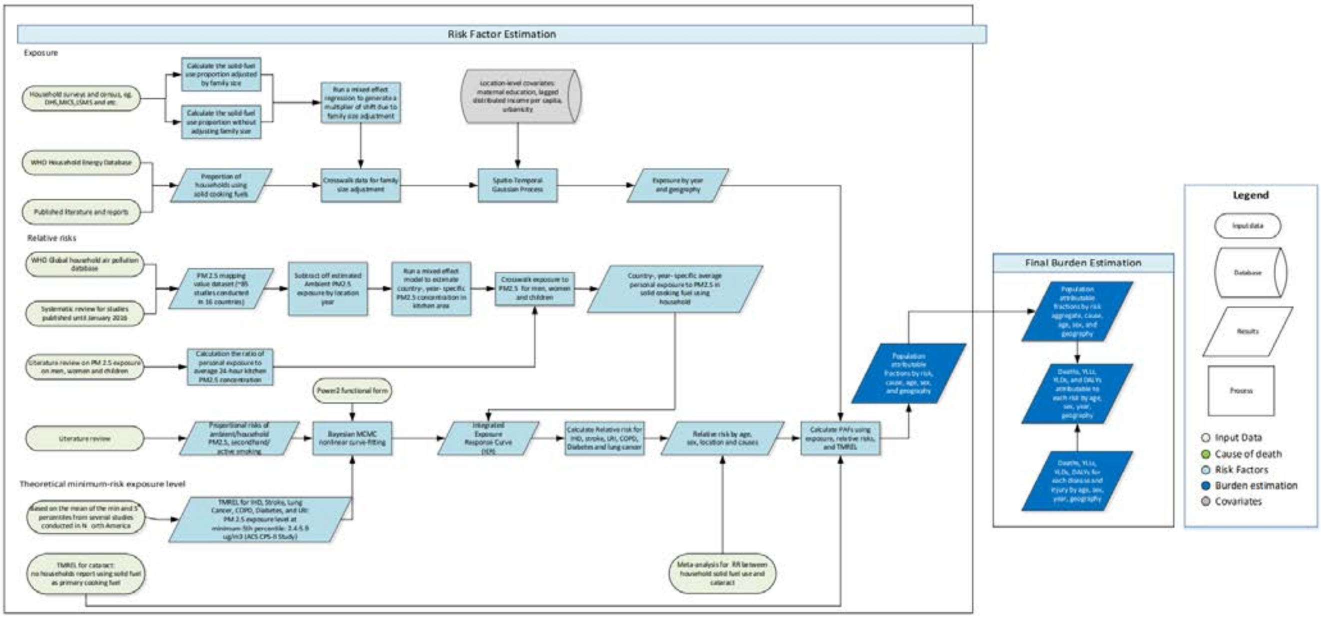

Exposure definition

Exposure to household air pollution from solid fuels (HAP) is defined as the proportion of households using solid cooking fuels. The definition of solid fuel in our analysis includes coal, wood, charcoal, dung, and agricultural residues.

Input data

Data were extracted from the standard multi-country survey series such as Demographic and Health Surveys (DHS), Living Standards Measurement Surveys (LSMS), Multiple Indicator Cluster Surveys (MICS), and World Health Surveys (WHS), as well as country-specific survey series such as Kenya Welfare Monitoring Survey and South Africa General Household Survey. To fill the gaps of data in surveys and censuses, we also downloaded and updated HAP estimates from WHO Energy Database and extracted from literature through systematic review. Each nationally or sub-nationally representative data point provided an estimate for the percentage of households using solid cooking fuels. Estimates for the usage of solid fuels for non-cooking purpose were excluded, i.e. primary fuels for lighting. The database, with estimates from 1980 to 2017, contained about 680 studies from 150 countries. As updates to systematic reviews are performed on an ongoing schedule across all GBD causes and risk factors, an update for household air pollution will be performed in the next 1-2 iterations.

The major data sources to measure this use in China included the China census[1-3], China Chronic Disease and Risk Factor Surveillance[4-6], China Energy Statistical Yearbook[7], China Health and Nutrition Survey[8.9], China National Health Services Survey[10-12], and scientific literature[13-17].

Modelling strategy

Household air pollution was modelled at household level using a three-step modelling strategy that uses linear regression, spatiotemporal regression and Gaussian Process Regression (GPR). The first step is a mixed-effect linear regression of logit-transformed proportion of households using solid

cooking fuels. The linear model contains maternal education, proportion of population living in urban areas, and lagged-distributed income as covariates and has nested random effect by GBD region, and GBD super region respectively. The full ST-GPR process is specified elsewhere this appendix. No substantial modelling changes were made in this round compared to GBD 2016.

Theoretical minimum-risk exposure level
For cataract, the TMREL is defined as no households using solid cooking fuel. For outcomes that utilise evidence based on the Integrated Exposure Response (IER), the TMREL is defined as uniform distribution between 2.4 and 5.9 ug/m^3.

Relative risks
In addition to the previously included outcomes of lower respiratory infections (LRI), stroke, IHD, COPD, lung cancer, and cataract, in GBD 2017 we added Type II Diabetes as a new outcome of household air pollution. The relative risk for cataracts was extracted from a meta-analysis and is 2.47 with 95% (1.61, 3.73).[18] GBD currently only estimates cataracts as an outcome for females. In GBD 2017, we adopted a new approach for risk attribution using the Integrated Exposure-Response Function (IER).

In order to use the IER curve, we must estimate the exposure to $PM_{2.5}$. Since GBD 2015 we have been using a mapping model relying on a database of now almost 90 studies which measures $PM_{2.5}$ exposure in households using solid cooking fuel. Using socio-demographic index and study-level factors as covariates, we predict exposure for all location-years. In GBD 2017, we updated the model to estimate the individual exposure to $PM_{2.5}$ over and above ambient levels due to the use of solid cooking fuel. We did this by subtracting off the estimated ambient level $PM_{2.5}$ for the location-year of each study in the database before inputting them into the model. By doing this we have independent estimates for $PM_{2.5}$ exposure due to ambient and household solid fuel use. These exposures are cross-walked to values for men, women, and children by generating the ratio of each group's mean exposure to the overall mean personal exposure. The resulting location, year, sex, and age specific $PM_{2.5}$ exposure values are used as inputs in the IER and attributable burden calculation process.

References

1. National Bureau of Statistics of China and Minnesota Population Center. China National Population Census 1990 from the Integrated Public Use Microdata Series, International: [Machine-readable database]. Minneapolis: University of Minnesota.
2. China Statistics Press, Minnesota Population Center. China National Population Census 2000 from the Integrated Public Use Microdata Series, International. Minneapolis: University of Minnesota, 2017.
3. National Bureau of Statistics of China. China Population and Housing Census 2010.
4. Chinese Center for Disease Control and Prevention (CCDC). China Chronic Disease and Risk Factor Surveillance 2004.
5. Chinese Center for Disease Control and Prevention (CCDC). China Chronic Disease and Risk Factor Surveillance 2007.
6. Chinese Center for Disease Control and Prevention (CCDC). China Chronic Disease and Risk Factor Surveillance 2010.

7. National Bureau of Statistics. China Energy Statistical Yearbook 2004-2017.
8. Carolina Population Center, University of North Carolina at Chapel Hill, Chinese Center for Disease Control and Prevention (CCDC). China Health and Nutrition Survey 1989. Chapel Hill, United States: Carolina Population Center, University of North Carolina at Chapel Hill.
9. Carolina Population Center, University of North Carolina at Chapel Hill, Chinese Center for Disease Control and Prevention (CCDC). China Health and Nutrition Survey 2004.
10. China Ministry of Health. China National Health Services Survey 1998.
11. China Ministry of Health. China National Health Services Survey 2003.
12. China Ministry of Health. China National Health Services Survey 2008.
13. Yu K, Qiu G, Chan K-H, et al. Association of solid fuel use with risk of cardiovascular and all-cause mortality in rural China. JAMA 2018; 319: 1351–61.
14. Kurmi OP, Arya PH, Lam K-BH, Sorahan T, Ayres JG. Lung cancer risk and solid fuel smoke exposure: a systematic review and meta-analysis. Eur Respir J 2012; 40: 1228–37.
15. Kim C, Seow WJ, Shu X-O, et al. Cooking coal use and all-cause and cause-specific mortality in a prospective cohort study of women in Shanghai, China. Environ Health Perspect 2016; 124: 1384–9.
16. Dherani M, Pope D, Mascarenhas M, Smith KR, Weber M, Bruce N. Indoor air pollution from unprocessed solid fuel use and pneumonia risk in children aged under five years: a systematic review and meta-analysis. Bull World Health Organ 2008; 86: 390–398C.
17. Fatmi Z, Coggon D. Coronary heart disease and household air pollution from use of solid fuel: a systematic review. Br Med Bull 2016; ll8: 91–109.
18. Smith KR, Bruce N, Balakrishnan K, Adair-Rohani H, Balmes J, Chafe Z, et al. Millions Dead: How Do We Know and What Does It Mean? Methods Used in the Comparative Risk Assessment of Household Air Pollution. Annu Rev Public Health. 2014;35(1):185–206.

Ambient ozone pollution

The steps in the estimation of disease burden attributable to ambient ozone pollution are shown in the following flowchart:

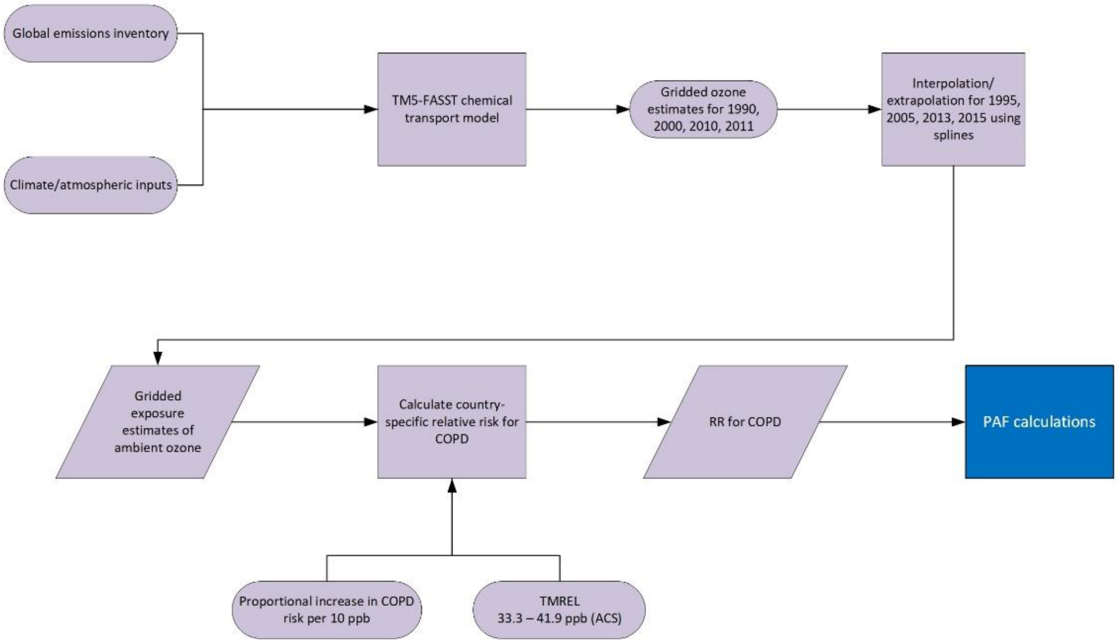

Exposure definition

For GBD 2017, exposure to ozone pollution is defined as the seasonal (6 month period with highest mean) 8 hour daily maximum ozone concentrations, measured in ppb. This was an update from the previous exposure metric in accordance with an update of the American Cancer Society Cancer Prevention Study II (ACS CPS-II).[1]

Input data

Previously, exposure estimates were based on a chemical transport model with no measurement database or evaluation. In GBD 2017, exposure estimates incorporated a new comprehensive ozone measurement database (TOAR).[2] This enabled a continent-specific weighted blend of 6 chemical transport models with grid cell level bias correction. The use of ground measurements also enabled the incorporation of error estimation, where previously we had assumed a +/- 6% error. The output of this model is a global raster of ozone exposure which is a summary for the years 2008-2014.[3]

Modelling strategy for trends

To estimate ozone concentrations over time, we used the trend from the former GBD model for 1990, 2000, and 2010 and cubic splines for 1995, 2005, and 2011, after applying an adjustment for the difference in trends between the previous (1 hour daily maximum) and current (8 hour daily maximum metrics. Annualised rate of change was used to predict for the years 2012-2017.

Theoretical minimum-risk exposure level

The TMREL of ozone was updated this year based on the exposure distribution from the updated ACS CPS-II study.[1] A uniform distribution was drawn around the minimum and 5th percentile values experienced by the cohort, defined as ~U(29.1, 35.7), in ppb.

Relative risks

Since the inclusion of ozone in GBD 2010, the relative risk of ozone exposure for COPD mortality has been defined to be 1.029, 95% CI. (1.01-1.048) per 10 ppb of ozone exposure. Note that this comes from one study that looked at all respiratory mortality.[4] For GBD 2017, we performed a literature review and included five cohorts from Canada, the UK, and the US which all measured COPD mortality. For cohorts with multiple analyses we chose the most recent analysis. We found a resulting relative risk of 1.06, 95% C.I. (1.02, 1.10).

Relative risk and proportional PAF approach

For GBD 2017 we developed a new approach to use the IER for obtaining PAFs for both OAP and HAP. Previously, relative risks for both exposures were obtained from the IER as a function of exposure and relative to the same TMREL. In reality, were a country to reduce only one of these risk factors, the other would remain. We failed to consider the joint effects of particulate matter from outdoor exposure and burning solid fuels for cooking.

In GBD 2017, relative risks were still estimated from the output of the IER curve. Everyone is exposed to some level of OAP, but only a proportion of the population in each location-year use solid cooking fuel and are exposed to HAP. For the proportion of the population not exposed to HAP the relative risk was obtained by $RR_{OAP}=IER(z= Exp_{OAP})$ and used to calculate the PAF for each location based on the population-weighted exposure. For the proportion of the population exposed to both OAP and HAP, we calculated a joint relative risk from the IER by $RR_{OAP+HAP}=IER(z= Exp_{OAP}+Exp_{HAP})$. This joint relative risk is used to calculate a joint PAF for each location. For each location, we proportioned the joint PAF based on the proportion of exposure due to OAP and HAP respectively. See the table below for equations used to calculate proportional PAFs.

PAF	Population not exposed to HAP	Population exposed to HAP
OAP	PAF_{OAP}	$(Exp_{OAP}/(Exp_{OAP}+Exp_{HAP}))*PAF_{OAP+HAP}$
HAP	0	$(Exp_{HAP}/(Exp_{OAP}+Exp_{HAP}))*PAF_{OAP+HAP}$

Generally, as expected, this new strategy led to lower PAFs for both ambient and household particulate matter pollution.

References

1. Turner MC,Jerrett M, Pope CA 3rd, Krewski D, Gapstur SM, Diver WR, Beckerman BS, Marshall JD, Su J, Crouse DL, Burnett RT. Long-term ozone exposure and mortality in a large prospective study. Am J Respir Crit Care Med. 2016; 193(10): 1134-42.

2. Schultz MG, Schröder S, Lyapina O, Cooper O, Galbally I, Petropavlovskikh I, et al.. Tropospheric Ozone Assessment Report: Database and Metrics Data of Global Surface Ozone Observations. Elem Sci Anth. 2017;5:58. DOI: http://doi.org/10.1525/elementa.244

3. Chang K-L, Cooper OR. A new method for combining observations and multiple model output for an improved estimate of the global surface ozone distribution. Atmospheric Chemistry and Physics Discussion, 2018.
4. Jerrett M, Burnett RT, Pope CA, et al. Long-term ozone exposure and mortality. N Engl J Med 2009; 360: 1085–95.

2.Use of solid fuels in provinces of China, 1990 and 2017; population weighted mean ambient ozone in provinces of China, 1990 and 2017.

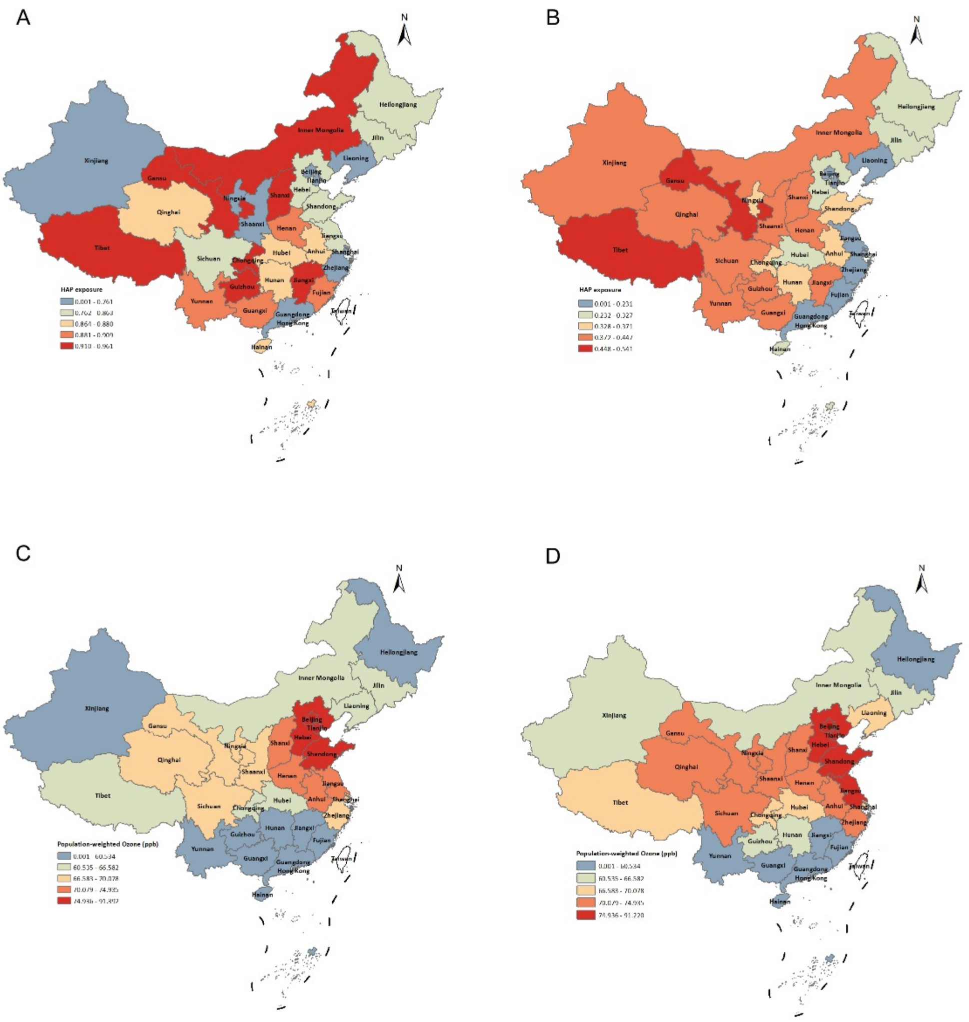

A: use of solid fuels in provinces of China, 1990

B: use of solid fuels in provinces of China, 2017

C: population weighted mean ambient ozone in provinces of China, 1990

D: population weighted mean ambient ozone in provinces of China, 2017.

3 Population weighted mean $PM_{2.5}$ levels, age-standardized death rate and DALY rate due to ambient $PM_{2.5}$ in China from 1990 to 2017.

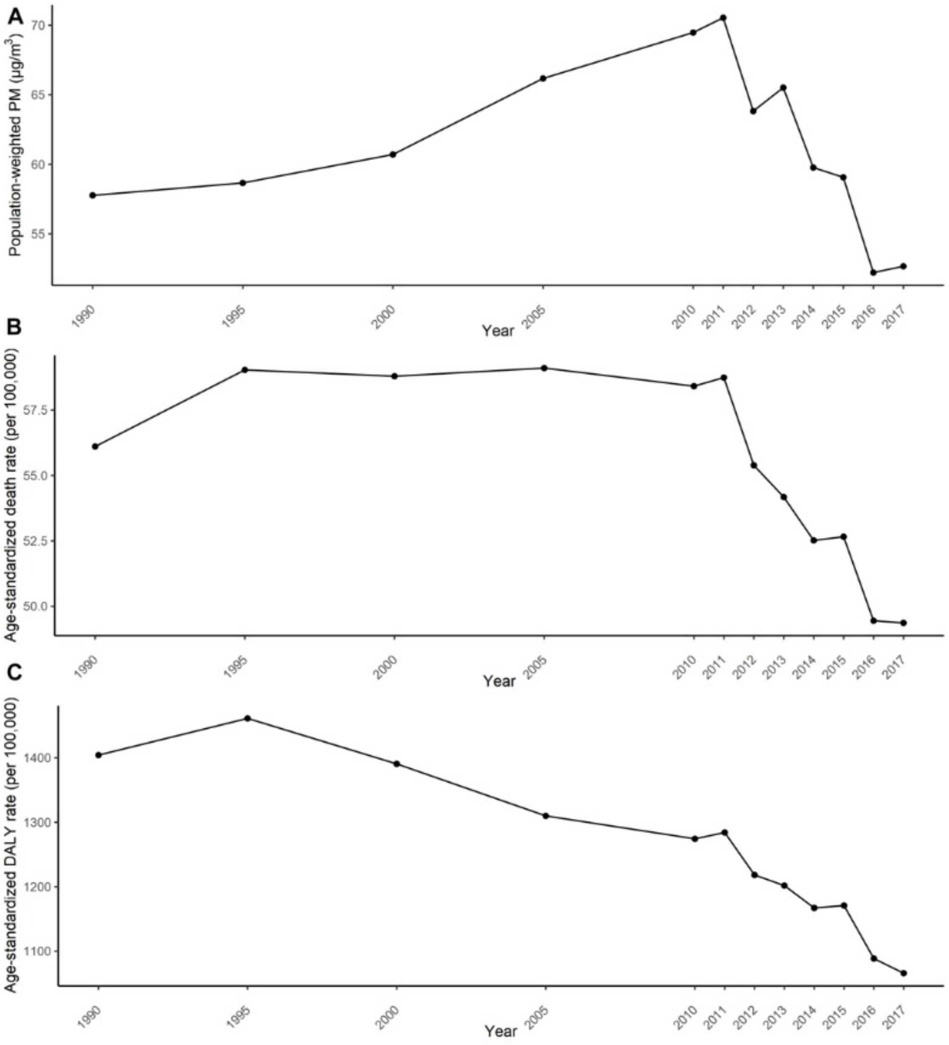

A: population weighted mean $PM_{2.5}$ levels from 1990 to 2017

B: age-standardized death rate due to ambient $PM_{2.5}$ in China from 1990 to 2017

C: age-standardized DALY rate due to ambient $PM_{2.5}$ in China from 1990 to 2017

4 Population Attributable Fractions (%) of DALYs for air pollution in all provinces in China, 2017

Province	Air pollution	$PM_{2.5}$	Household air pollution	Ambient ozone pollution
**China**	6·87 (5·93-7·82)	4·84 (4·04-5·70)	1·61 (1·26-2·06)	0·63 (0·23-1·02)
**Health area 1**				
Shanghai	4·39 (3·68-5·18)	4·03 (3·34-4·78)	0·12 (0·06-0·21)	0·35 (0·14-0·59)
Tianjin	7·88 (6·82-9·04)	7·32 (6·25-8·50)	0·31 (0·16-0·53)	0·39 (0·15-0·66)
Zhejiang	4·90 (3·99-5·91)	4·05 (3·15-5·00)	0·46 (0·25-0·78)	0·55 (0·21-0·90)
Beijing	5·90 (4·95-6·92)	5·52 (4·58-6·54)	0·14 (0·07-0·26)	0·37 (0·15-0·60)
Hong Kong	4·16 (3·37-4·97)	3·26 (2·51-4·03)	0·81 (0·44-1·33)	0·11 (0·04-0·22)
Macau	4·52 (3·67-5·39)	3·48 (2·72-4·26)	0·91 (0·50-1·42)	0·18 (0·06-0·34)
**Health area 2**				
Jiangsu	6·22 (5·20-7·30)	5·00 (3·99-6·01)	0·69 (0·40-1·14)	0·78 (0·30-1·23)
Hainan	4·77 (3·88-5·67)	2·51 (1·70-3·37)	2·03 (1·35-2·80)	0·31 (0·11-0·53)
Guangdong	5·44 (4·53-6·49)	4·54 (3·60-5·56)	0·66 (0·40-1·06)	0·32 (0·11-0·54)
Fujian	4·52 (3·73-5·40)	3·43 (2·72-4·24)	0·81 (0·50-1·25)	0·38 (0·14-0·64)
Hubei	6·92 (5·79-8·13)	5·04 (3·85-6·36)	1·42 (0·84-2·15)	0·69 (0·26-1·11)
Hunan	7·42 (6·27-8·67)	4·98 (3·76-6·30)	1·92 (1·20-2·79)	0·77 (0·28-1·25)
**Health area 3**				
Shandong	7·53 (6·19-8·83)	5·87 (4·25-7·22)	1·21 (0·73-1·91)	0·69 (0·26-1·13)
Hebei	7·52 (6·04-8·77)	6·01 (3·98-7·50)	1·15 (0·62-1·99)	0·58 (0·22-0·96)
Ningxia	6·58 (5·61-7·56)	4·25 (3·29-5·28)	1·93 (1·33-2·73)	0·57 (0·22-0·92)
Jilin	6·83 (5·77-8·07)	5·16 (3·85-6·52)	1·53 (0·87-2·33)	0·21 (0·07-0·46)
Liaoning	7·20 (6·14-8·40)	5·99 (4·78-7·40)	1·01 (0·55-1·71)	0·28 (0·10-0·59)
Shanxi	6·74 (5·66-7·87)	4·74 (3·50-5·95)	1·67 (1·09-2·47)	0·49 (0·19-0·85)
Shaanxi	6·42 (5·37-7·59)	4·38 (3·07-5·62)	1·76 (1·16-2·62)	0·43 (0·16-0·73)
Henan	8·02 (6·91-9·20)	5·88 (4·55-7·15)	1·78 (1·11-2·71)	0·56 (0·21-0·97)
Anhui	6·40 (5·41-7·46)	4·27 (3·11-5·33)	1·76 (1·20-2·46)	0·58 (0·22-0·95)
Inner Mongolia	6·94 (5·73-8·17)	4·29 (2·70-5·75)	2·28 (1·35-3·40)	0·53 (0·19-0·87)
Heilongjiang	7·32 (5·86-8·90)	5·29 (3·37-7·22)	1·81 (1·03-2·86)	0·32 (0·12-0·61)
**Health area 4**				
Jiangxi	6·55 (5·56-7·64)	3·73 (2·72-4·77)	2·42 (1·67-3·36)	0·59 (0·21-0·99)
Chongqing	7·47 (6·16-8·86)	4·94 (3·80-6·21)	1·78 (1·21-2·57)	1·11 (0·41-1·81)
Yunnan	6·93 (5·70-8·17)	3·24 (2·09-4·54)	3·18 (2·29-4·18)	0·72 (0·25-1·20)
Gansu	7·61 (6·38-8·85)	3·62 (2·48-4·92)	3·24 (2·35-4·29)	1·13 (0·42-1·80)
Sichuan	7·73 (6·12-9·13)	4·75 (2·70-6·24)	2·14 (1·37-3·22)	1·24 (0·47-1·99)
**Health area 5**				
Tibet	7·97 (6·71-9·40)	2·01 (0·98-3·78)	5·27 (3·98-6·49)	1·00 (0·37-1·62)
Xinjiang	8·88 (6·59-11·12)	6·09 (2·52-9·19)	2·26 (1·09-3·83)	0·82 (0·30-1·36)
Qinghai	7·98 (6·54-9·30)	4·46 (2·38-5·94)	2·76 (1·88-4·04)	1·11 (0·42-1·80)

Guangxi	6·25 (5·30-7·23)	3·85 (2·89-5·00)	2·11 (1·50-2·87)	0·42 (0·15-0·69)
Guizhou	7·46 (6·31-8·68)	3·52 (2·52-4·79)	3·38 (2·48-4·40)	0·82 (0·30-1·35)

5. Percentage and 95% UI of DALYs attributable to air pollution for different diseases in China, 2017

Disease	Percentage, 95%UI
COPD	40.0% (29.4%-49.3%)
Diabetes mellitus	26.1% (28.4%-42.5%)
Ischemic heart disease	19.5% (17.4%-21.8%)
Lower respiratory infections	35.6% (28.4%-42.5%)
Stroke	12.8% (17.4%-21.8%)
Lung cancer	25.8% (19.1%-32.5%)

6. DALYs (in thousand) attributable to air pollution with 95% uncertainty interval of China in 2017

	Air pollution	Ambient particulate matter pollution	Household air pollution from solid fuels	Ambient ozone pollution
**All causes**	27934·1 (24391·0-31198·5)	19804·9 (16515·0-22947·5)	6463·9 (5095·3-8211·9)	2468·0 (938·8-3972·0)
**Disease**				
Blindness and vision impairment	174·8 (81·6-288·8)	-	174·8 (81·6-288·8)	-
COPD	8160·7 (6019·8-10155·3)	4794·5 (3088·9-6270·9)	1700·9 (1080·3-2413·4)	2468·0 (938·8-3972·0)
Diabetes mellitus	2615·9 (1645·3-3513·2)	2020·7 (1249·7-2712·3)	595·2 (342·1-856·3)	-
Ischemic heart disease	5868·1 (5221·8-6610·5)	4506·4 (3812·3-5231·2)	1361·6 (1056·9-1757·9)	-
Lower respiratory infections	1529·8 (1189·0-1942·7)	1091·8 (822·3-1440·1)	438·0 (327·0-565·4)	-
Stroke	5647·6 (4546·3-6757·6)	4290·9 (3342·1-5264·5)	1356·8 (985·3-1813·2)	-
Lung cancer	3937·2 (2887·0-4939·9)	3100·5 (2238·0-3976·3)	836·7 (563·2-1141·9)	-
**Sex**				
Male	16711·4 (14574·3-18722·0)	12493·5 (10574·9-14449·5)	3193·2 (2326·8-4230·3)	1507·0 (572·2-2423·3)
Female	11222·8 (9721·5-12735·0)	7311·3 (6007·5-8606·6)	3270·8 (2604·0-4107·2)	961·0 (360·5-1602·9)
**Age**				
Children<5 years	670·3 (518·8-822·1)	454·4 (332·8-584·2)	215·8 (155·3-285·1)	0·00 (0·00-0·00)
Elderly>70 years	10773·8 (9182·5-12292·3)	7230·2 (5892·5-8543·2)	2456·1 (1910·9-3140·8)	1611·8 (612·4-2585·6)

DALY=disability-adjusted life-year·

7. Age-standardized death rate and DALY rate attributable to air pollution of China, 1990 and 2017 with percentage changes

	Age-standardized death rate, per 100,000			Age-standardized DALY rate, per 100,000		
	1990	2017	Percentage changes (%)	1990	2017	Percentage changes (%)
**Air pollution**						
All causes	184·4 (161·4-205·6)	72·7 (63·2-81·5)	-60·6 (-63·7--55·7)	4597·4 (4067·2-5096·6)	1513·1 (1324·9-1688·4)	-67·1 (-70·1--63·8)
Blindness and vision impairment				18·5 (10·3-28·0)	10·0 (4·7-16·6)	-45·7 (-55·9--37·3)
COPD	101·2 (78·6-119·5)	26·8 (19·4-33·8)	-73·5 (-76·9--67·0)	1679·9 (1323·5-1971·7)	449·4 (330·7-558·7)	-73·3 (-76·4--67·9)
Diabetes mellitus	1·1 (0·8-1·4)	1·8 (1·2-2·1)	62·6 (37·4-109·8)	115·0 (76·1-157·7)	134·5 (84·7-181·2)	16·9 (4·6-26·9)
Ischemic heart disease	18·1 (16·3-20·1)	16·6 (14·7-18·8)	-7·9 (-17·0--0·3)	375·8 (340·7-416)	304·9 (271·8-342·0)	-18·9 (-26·4--12·2)
Lower respiratory infections	27·4 (21·9-32·0)	4·6 (3·6-5·9)	-83·3 (-86·0--78·1)	1600·1 (1284·3-1898·1)	131·1 (101·9-165·0)	-91·8 (-93·1--89·8)
Stroke	26·0 (21·6-30·3)	13·5 (10·7-16·2)	-48·1 (-52·8--43·9)	556·8 (471·4-646·9)	287·8 (233·6-343·6)	-48·3 (-52·9--44·2)
Lung cancer	10·7 (8·5-12·6)	9·4 (6·9-11·8)	-11·8 (-24·6--1·0)	251·3 (200·2-297·6)	195·4 (143·2-245·0)	-22·3 (-33·7--12·5)
**Ambient particulate matter pollution**						
All causes	56·1 (43·1-67·5)	49·4 (41·2-57·5)	-12·0 (-22·1-1·4)	1404·2 (1088·7-1690·4)	1065·9 (891·3-1237·4)	-24·1 (-33·2--12·5)
COPD	27·5 (19·0-35·4)	14·2 (9·3-18·7)	-48·2 (-57·0--35·8)	471·2 (329·5-607·4)	263·1 (170·0-344·4)	-44·2 (-53·2--32·9)
Diabetes mellitus	0·4 (0·3-0·5)	1·4 (0·9-1·6)	237·8 (175·3-346·7)	42·2 (26·4-60·0)	104·0 (64·2-139·8)	146·6 (112·8-188·8)
Ischemic heart disease	6·6 (5·3-7·9)	12·7 (10·7-14·7)	93·5 (69·9-122·3)	138·0 (110·0-164·8)	234·1 (200·2-269·1)	69·6 (49·7-93·8)
Lower respiratory infections	8·3 (5·9-10·5)	3·4 (2·5-4·5)	-59·4 (-66·6--46·4)	458·3 (322·4-590·1)	92·1 (68·5-119·0)	-79·9 (-83·9--73·9)
Stroke	9·3 (7·0-11·6)	10·2 (7·8-12·5)	10·5 (-2·5-25·7)	198·5 (150·7-249·9)	218·7 (171·2-268·0)	10·2 (-2·2-25·6)
Lung cancer	4·1 (3·1-5·1)	7·4 (5·4-9·5)	81·5 (53·1-109·9)	96·0 (72·6-120·4)	153·9 (111·1-197·1)	60·4 (35·1-85·5)
**Household air pollution from solid fuels**						
All causes	108·0 (89·9-126·2)	15·8 (12·2-20·2)	-85·4 (-87·3--83·2)	2907·5 (2465·6-3348·8)	354·0 (279·4-448·2)	-87·8 (-89·5--85·9)
Blindness and vision impairment	53·4 (38·8-66·6)	5·0 (3·2-7·1)	-90·5 (-92·3--88·0)	18·5 (10·3-28·0)	10·0 (4·7-16·6)	-45·7 (-55·9--37·3)
COPD	0·7 (0·5-0·9)	0·4 (0·3-0·5)	-40·4 (-52·8--18·9)	923·0 (679·4-1149·7)	93·1 (59·2-132·1)	-89·9 (-91·7--87·7)
Diabetes mellitus	11·5 (9·7-13·5)	3·9 (3·0-5·0)	-66·0 (-71·1--60·8)	72·9 (46·9-101·5)	30·5 (17·6-43·9)	-58·1 (-65·0--50·4)
Ischemic heart disease	19·1 (15·0-23·0)	1·2 (0·9-1·6)	-93·6 (-94·8--92·0)	237·8 (201·4-278·1)	70·8 (55·1-91)	-70·2 (-74·4--65·8)
Lower respiratory infections	16·7 (13·5-20·2)	3·2 (2·4-4·3)	-80·6 (-83·5--77·9)	1141·8 (894·2-1378)	39·0 (29·0-51·2)	-96·6 (-97·2--95·8)
Stroke	6·6 (5·0-8·2)	2·0 (1·3-2·7)	-69·8 (-74·5--65·0)	358·3 (289·8-432·5)	69·1 (50·7-92·1)	-80·7 (-83·5--78·1)
Lung cancer	108·0 (89·9-126·2)	15·8 (12·2-20·2)	-85·4 (-87·3--83·2)	155·3 (118·6-194·3)	41·5 (27·9-56·6)	-73·3 (-77·5--69·0)

**Ambient ozone pollution**						
COPD	35·3 (13·5-56·6)	11·1 (4·2-17·8)	-44·3 (-48·4--32·5)	495·1 (190·0-796·3)	138·1 (52·4-222·0)	-72·1 (-74·2--66·1)

8. Comparison of age-standardized death rate and DALY rate attributable to air pollution in 2013 and 2017 in China

	Age-standardized death rate, per 100,000		Age-standardized DALY rate, per 100,000	
	2013	2017	2013	2017
**Air pollution**				
All causes	81.7 (72.0-91.5)	72.7 (63.2-81.5)	1763.6 (1564.1-1962.6)	1513.1 (1324.9-1688.4)
Blindness and vision impairment	-	-	13.3 (6.5-21.5)	10.0 (4.7-16.6)
COPD	30.4 (22.8-38.0)	26.8 (19.4-33.8)	528.9 (392.4-650.9)	449.4 (330.7-558.7)
Diabetes mellitus	1.7 (1.2-2.0)	1.8 (1.2-2.1)	141.3 (90.1-188.5)	134.5 (84.7-181.2)
Ischemic heart disease	18.4 (16.3-20.5)	16.6 (14.7-18.8)	335.1 (300.8-372.8)	304.9 (271.8-342.0)
Lower respiratory infections	6.1 (4.7-7.5)	4.6 (3.6-5.9)	220.0 (173.1-267.9)	131.1 (101.9-165.0)
Stroke	15.0 (12.2-17.7)	13.5 (10.7-16.2)	313.6 (257.5-369.1)	287.8 (233.6-343.6)
Lung cancer	10.1 (7.7-12.5)	9.4 (6.9-11.8)	211.4 (159.7-261.1)	195.4 (143.2-245.0)
**Ambient particulate matter pollution**				
All causes	54.2 (45.1-63.2)	49.4 (41.2-57.5)	1201.8 (1001.1-1395.0)	1065.9 (891.3-1237.4)
COPD	16.2 (11.0-21.1)	14.2 (9.3-18.7)	309.3 (210.0-400.9)	263.1 (170.0-344.4)
Diabetes mellitus	1.3 (0.9-1.5)	1.4 (0.9-1.6)	105.9 (66.8-143.1)	104.0 (64.2-139.8)
Ischemic heart disease	13.6 (11.5-15.7)	12.7 (10.7-14.7)	249.0 (209.9-285.7)	234.1 (200.2-269.1)
Lower respiratory infections	4.2 (3.2-5.5)	3.4 (2.5-4.5)	145.3 (110.2-185.0)	92.1 (68.5-119.0)
Stroke	11.0 (8.6-13.4)	10.2 (7.8-12.5)	230.6 (182.0-281.3)	218.7 (171.2-268.0)
Lung cancer	7.8 (5.8-9.8)	7.4 (5.4-9.5)	161.7 (120.4-205.0)	153.9 (111.1-197.1)
